# LSWOA: An enhanced whale optimization algorithm with Levy flight and Spiral flight for numerical and engineering design optimization problems

**DOI:** 10.1371/journal.pone.0322058

**Published:** 2025-09-03

**Authors:** Junhao Wei, Yanzhao Gu, Zhanxi Xie, Yuzheng Yan, Baili Lu, Zikun Li, Ngai Cheong, Jiafeng Zhang, Song Zhang

**Affiliations:** 1 Faculty of Applied Sciences, Macao Polytechnic University, Macao, China; 2 Faculty of Humanities and Social Sciences, Macao Polytechnic University, Macao, China; 3 College of Animal Science and Technology, Zhongkai University of Agriculture and Engineering, Guangzhou, China; 4 School of Economics and Management, South China Normal University, Guangzhou, China; 5 Avaition College, Beijing Institute of Technology (Zhuhai), Zhuhai, China; Torrens University Australia, AUSTRALIA

## Abstract

Whale Optimization Algorithm (WOA) suffers from issues such as premature convergence, low population diversity in the later stages of iteration, slow convergence rate, low convergence accuracy, and an imbalance between exploration and exploitation. Thus, an enhanced Whale Optimization Algorithm (LSWOA) based on multiple strategies is proposed, aiming to overcome the limitations of the canonical WOA. The performance of the canonical WOA is improved through innovative strategies: first, an initialization process using Good Nodes Set is introduced to ensure that the search starts from a higher-quality baseline; second, a distance-based guided search strategy is employed to adjust the search direction and intensity by calculating the distance to the optimal solution, which enhances the algorithm’s ability to escape local optima; and lastly, LSWOA introduces an enhanced spiral updating strategy, while the enhanced spiral-enveloping prey strategy effectively balances exploration and exploitation by dynamically adjusting the spiral shape parameters to adapt to different stages of the search, thereby more accurately updating the positions of individuals and improving convergence speed. In the experimental section, we validate the efficiency and superiority of LSWOA by comparing it with outstanding metaheuristic algorithms and excellent WOA variants. The experimental results show that LSWOA exhibits significant optimization performance on the benchmark functions with various dimensions. Additionally, LSWOA is tested on seven engineering design optimization problems, and the results demonstrate that it performs excellently in these application scenarios, effectively solving complex optimization problems in different dimensions and showing its potential for a wide range of applications in real-world engineering challenges.

## Author summary

I received my B.S. degree in Unmanned Aerial Vehicle System Engineering from Aviation College, Beijing Institute of Technology (Zhuhai) in 2023. I’m now pursuing a M.S. degree in Big Data and Internet of Things at Macao Polytechnic University. I have strong interest in metaheuristic algorithms, RRT algorithm and APF algorithm. During my MSc at Macao Polytechnic University, I worked as a research assistant, mainly responsible for the research work of UAV path planning.

## 1 Introduction

Research on metaheuristic algorithms began in the late 20th century. With the development of computer science, more researchers have focused on utilizing intelligent algorithms to solve complex optimization problems. Metaheuristic algorithms are high-level heuristic methods that aim to find the global optimal solution by simulating natural phenomena or biological behavior. These algorithms combine random search and local search strategies to effectively avoid the problem of getting stuck in local optima, which is a common challenge for canonical algorithms. The origin of metaheuristic algorithms can be traced back to the 1960s, with the development of genetic algorithms and simulated annealing. Since the 21st century, numerous metaheuristic algorithms have been developed and applied: Harris Hawks Optimization (HHO) [1], Sparrow Search Algorithm (SSA) [2], Butterfly Optimization Algorithm (BOA) [3], Chimp Optimization Algorithm (ChOA) [4], Crayfish Optimization Algorithm (COA) [5], Bat Algorithm (BA) [6], and Greater Cane Rat Algorithm (GCRA) [7], among others.

Metaheuristic algorithms have been widely used in various fields due to their powerful optimization capabilities. These applications include neural network tuning [8], feature selection [9], urban planning [10], path planning [11], antenna design optimization [12] [13], workshop scheduling [14], power distribution optimization [15], and more. Metaheuristic algorithms effectively handle complex multi-objective and multi-constraint problems, greatly improving decision-making efficiency. For instance, in logistics distribution, Ant Colony Optimization (ACO) algorithm efficiently finds optimal paths for multiple distribution points, reducing transportation costs [16]. In network planning, Particle Swarm Optimization(PSO) helps optimize routing decisions, minimizing network latency [17]. In intelligent traffic systems, metaheuristic algorithms are used to optimize traffic light settings, alleviating congestion [18]. These applications not only demonstrate the theoretical advantages of metaheuristic algorithms but also drive their widespread use in real-world applications. However, when metaheuristic algorithms were applied in real life, researchers found that they also have certain limitations. These algorithms often face challenges such as the difficulty in balancing exploration and exploitation, difficulties in parameter selection, poor population quality in the later stages of iteration, and the tendency to become trapped in local optima. Particle Swarm Optimization (PSO) faces the risk of premature convergence when dealing with complex optimization problems, often leading to early convergence to local optima, halting further exploration of better solutions. The Harris Hawks Optimization (HHO) algorithm is known for its strong exploration and exploitation capabilities, but its numerous parameters and complex position update strategies make its tuning more difficult. The Whale Optimization Algorithm (WOA) is simple in structure and easy to implement, but it suffers from slow convergence, low solution accuracy, and the tendency to get trapped in local optima in complex problems [30]. Meanwhile, WOA performs poor in solving most engineering design problems. Therefore, improving the balance between exploration and exploitation, enhancing the efficiency of the exploration phase, increasing the accuracy of the exploitation phase, and maintaining population diversity in the later stages of iteration have become the major challenges in enhancing the performance of metaheuristic algorithms.

As metaheuristic algorithms have been applied more deeply in various fields, researchers have continuously sought to improve them to further enhance performance. Various improvement strategies have been proposed to increase the precision of search and convergence speed, such as new population initialization methods, hybrid algorithms, mutation strategies, and adaptive parameter adjustments. In 2005, B Liu *et al*. proposed a chaotic PSO (CPSO) [32]. CPSO introduced chaotic mapping into population initialization, improving the optimization capability of PSO. Hybrid algorithms combine the advantages of different algorithms to compensate for the weaknesses of the original ones. For example, in 2022, Yaning Xiao *et al*. combined Aquila Optimization (AO) algorithm with the African Vulture Optimization Algorithm (AVOA), proposed IHAOAVOA [20]. IHAOAVOA, which overcame the deficiencies in the single algorithm and provided higher-quality solutions for solving global optimization problems, could enhance both global and local search capabilities. Mutation strategies introduce random disturbances, such as Gaussian mutation and Cauchy mutation, to prevent the algorithm from falling into local optima. In 2003, Higashi N *et al*. proposed PSO_with_Gaussian_Mutation, which incorporated the idea of Genetic Algorithm (GA) and Gaussian mutation. PSO_with_Gaussian_Mutation (GPSO) performed better than standard PSO and standard GA [22]. In 2007, Wang H *et al*. introduced Gaussian mutation into standard PSO (HPSO) [21]. Results shown that HPSO could successfully deal with those difficult multimodal functions while maintaining fast search speed on those simple unimodal functions. Adaptive parameter adjustment dynamically modifies algorithm parameters based on feedback during the optimization process, improving adaptability. These improvements have not only achieved remarkable academic research results but have also shown great potential in practical engineering applications.

Whale Optimization Algorithm (WOA) is a nature-inspired metaheuristic algorithm developed by Mirjalili *et al*. in 2016, which mimics the hunting strategy of humpback whales [30]. It has strong global optimization capabilities and a simple structure. However, WOA also has certain drawbacks, such as the tendency to get trapped in local optima, low convergence precision, and difficulty in balancing global exploration and local exploitation. These years, researchers tried to enhance WOA with various strategies. In 2019, H Chen *et al*. proposed an improved Whale Optimization Algorithm (BWOA), which integrates Levy Flight and chaotic local search strategy, aiming to enhance its search capability in optimization tasks [23]. In 2021, S Chakrabort *et al*. proposed a novel enhanced WOA (WOAmM) which incorporated the mutualistic symbiosis phase in SOS Algorithm, to enhance the exploration ability of the search space and help avoid the waste of computational resources caused by overexploitation [24]. In 2022, Lin X *et al*. proposed a heuristic WOA with niching strategy (NHWOA), which integrates the niche strategy, Levy Flight and subpopulation distribution strategy, improving optimization performance through adaptive updates and local searches at different stages [25]. In 2022, Yang W *et al*. proposed a multi-strategy WOA (MSWOA), which integrates dynamic weight updating, local search strategies, Levy Flight and elitism preservation to enhance optimization efficiency and global search capability [36]. However, although these algorithms strike a good balance between exploration and exploitation, they fail to effectively balance convergence speed and accuracy. And they are not effective in maintaining high population diversity. Therefore, this paper proposed an enhanced WOA based on multiple strategies (LSWOA) to address the above issues.

LSWOA used Good Nodes Set initialization to generate a more uniform distribution of the population, incorporated Distance-Guided Prey Searching (DGPS) strategy to perform a better exploration, incorporated Spiral Encircling Prey (SEP) strategy and Enhanced Spiral Updating strategy to help escaping local optima and adopted a newly-designed update method of convergence factor *a* to better balance exploration and exploitation, aiming to address the limitations of original WOA. Experiments show that LSWOA not only struck a good balance between exploration and exploitation, but also ensured good convergence. In engineering design optimization simulation, LSWOA demonstrated strong global search capability and detailed local development ability, with a fast convergence rate. The balance between exploration and exploitation was well maintained. Table 1 is the current research on improved metaheuristic algorithms.

**Table 1:** Current research on improved metaheuristic algorithms.

Algorithm	Year	Author	Source of Inspiration
CPSO [19]	2005	Liu B *et al*.	Adaptive inertia weight factor and
			chaotic searching.
IHAOAVOA [20]	2022	Y Xiao *et al*.	Exploration of Aquila Optimizer (AO)
			and exploitation of African Vultures
			Optimization Algorithm (AVOA).
GPSO [22]	2003	N Higashi *et al*.	Genetic Algorithm (GA) and Gaussian
			mutation.
HPSO [21]	2007	H Wang *et al*.	Cauchy mutation.
BWOA [23]	2019	H Chen *et al*.	Levy flight and chaotic local search.
WOAmM [24]	2020	S Chakraborty *et al*.	Symbiotic Organisms Search (SOS).
NHWOA [25]	2022	Liu M *et al*.	Levy flight and Differential
			Evolution (DE).
MSWOA [36]	2022	W Yang *et al*.	Adaptive inertia weight, dynamic
			convergence factor and Levy flight.

## 2 Structure of the paper

The remaining sections of this paper are organized as follows: Chapter 3 briefly Ooverviewed the major contribution of this research. Section 4 introduces the current research works on engineering design. Section 5 introduces the principles of WOA and its advantages and disadvantages. Section 6 presents the proposed improved WOA based on multiple strategies (LSWOA). Section 7 evaluates the performance of the LSWOA by six experiments. Section 8 discusses its application to real-world engineering design optimization problems.

## 3 Major contributions

The structure of WOA is relatively simple, making it easy to understand and implement. However, WOA struggles to balance exploration and exploitation, and the population quality tends to deteriorate significantly over iterations, leading to insufficient global exploration and premature convergence to local optima. Although the aforementioned studies mentioned in Chapter 1 have improved the performance of WOA to some extent, most of them fail to simultaneously balance exploration and exploitation, enhance convergence speed and accuracy, effectively escape local optima, and maintain a high level of population diversity in the later stages of iteration. Considering WOA performs poor in engineering optimization design, we proposed an enhanced whale algorithm with multi-strategy (LSWOA). LSWOA aimed to make up for the shortcomings of WOA to a certain extent and explore the potential of WOA as an excellent optimizer for engineering design optimization.

LSWOA introduced Good Nodes Set Initialization to generate uniformly distributed populations, employs a newly designed Distance-Guided Prey Searching strategy to enhance global exploration, incorporated Spiral Encircling Prey strategy that integrates Spiral flight, utilized an Enhanced Spiral Updating Strategy with Levy flight and inertia weight ω, and introduced a new update mechanism for the convergence factor *a* to better balance exploration and exploitation. Experiments showed that LSWOA effectively addresses the drawbacks of WOA. Furthermore, compared to the classical WOA and other state-of-the-art (SOTA) metaheuristic algorithms, LSWOA demonstrated significant advantages in both numerical optimization and real-world optimization problems.

## 4 Research works on engineering design

Nowadays, as the rapid development of metaheuristic algorithms, they have been particularly widely applied in engineering design optimization [27]. Since the early 21st century, metaheuristic algorithms have been introduced to various engineering optimization problems. Before this, engineering design heavily relied on engineers’ experience and intuition. Although some numerical optimization methods were introduced, they were often limited by problem complexity and struggled to find global optima. With the rapid development of computer technology and the maturation of metaheuristic algorithms, engineering design optimization entered a new era. For instance, in pressure vessel design, canonical designs relied on experience and experimentation [28]. By introducing metaheuristic algorithms, multiple parameters such as vessel size and materials can be optimized for multi-objective goals, significantly reducing material costs while ensuring safety. In rolling bearing design [29], metaheuristic algorithms optimize parameters such as geometric dimensions and contact angles to achieve longer bearing life and higher load capacity.

Compared to canonical engineering design methods, metaheuristic algorithms offer several advantages. First, they can efficiently handle complex optimization problems, such as high-dimensional, multi-constraint, and nonlinear problems, without relying on specific mathematical models. Second, metaheuristic algorithms possess strong global search capabilities, allowing them to escape local optima and find global solutions. Additionally, these algorithms exhibit good robustness, maintaining high optimization performance across different application scenarios. These advantages have made metaheuristic algorithms important tools in modern engineering design and effective solutions for complex optimization problems. This paper will explore the effectiveness and applicability of LSWOA in engineering design optimization, aiming to provide a new optimizer for engineering design optimization.

## 5 WOA

The Whale Optimization Algorithm (WOA), proposed by Mirjalili *et al*. in 2016, is a metaheuristic optimization algorithm inspired by the hunting behavior of humpback whales [30]. In WOA, the spiral upward strategy and encircling prey strategy of humpback whales are simulated.

### 5.1 Encircling prey

Humpback whales can identify the location of their prey and encircle them. Since the optimal location in the search space is unknown, WOA assumes that the current best candidate solution is the prey or close to the optimal solution. Once the best search agent is defined, other agents attempt to update their positions toward the best search agent. This behavior is described by Eqs 1 and 2.

$$D=|C\cdot X^{*}(t)-X(t)|$$ (1)

$$X(t+1)=X^{*}(t)-A\cdot D$$ (2)

where *t* is the current iteration; *A* and *C* are coefficient vectors; ${\text{X}}^{*}(\text{t})$ is the position of the current best solution; *X*(*t*) is the position of the whale.

The results of each iteration are updated if a better solution is found, and the corresponding fitness value improves.

The coefficients *A* and *C* are calculated as follows:

A=2a·r−a (3)

$$a=2-2\cdot \frac{t}{T}$$ (4)

**Fig 1:**
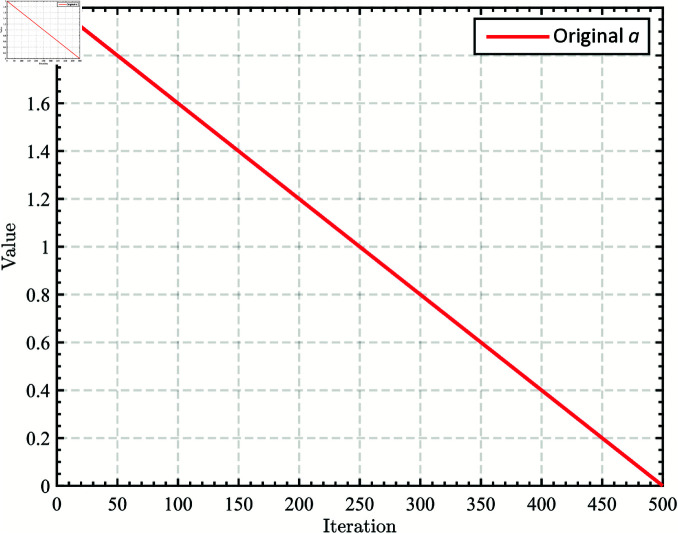
The linearly decreasing convergence factor a.

C=2·r (5)

where *r* is a random number between 0 and 1; *a* is the convergence factor, which decreases linearly from 2 to 0 over the course of iterations, as shown in Fig 1.

### 5.2 Bubble-net attacking method

In addition to encircling prey, whales use a bubble-net attacking method to trap prey by spiraling upward while creating bubbles. This strategy involves two main mechanisms: shrinking encircling and spiral updating.

#### 5.2.1 Shrinking encircling.

This behavior is modeled by decreasing the value of *A* in Eq 4.

#### 5.2.2 Spiral updating.

The distance between the whale and prey is calculated by Eq 6, and a spiral equation simulates the upward spiral motion to encircle prey as Eq 9.

$$X(t+1)=X^{*}(t)+D^{\prime }\cdot e^{bl}\cdot \cos(2\pi l)$$ (6)

$$D^{\prime }=|X^{*}(t)-X(t)|$$ (7)

$$l=(a_{1}-1)\cdot Rand+1$$ (8)

$$a_{1}=-1-\frac{t}{T}$$ (9)

where *b* is a constant that defines the shape of a logarithmic spiral, usually set to 1; *a*_1_ is the parameter of the linear change of [–2,–1]; *Rand* is a random number between 0 and 1; the value of spiral coefficient *l* is [–2,1].

When the WOA algorithm sets the whale’s update position, the encircling strategy and the spiral update strategy each have a 50% probability, that is:

$$X(t+1)=\left\{ \begin{array}{cc} X^{*}(t)-A\cdot D & p<0.5\\ X^{*}(t)+D^{\prime }\cdot e^{bl}\cdot cos(2\pi l) & p\geq 0.5 \end{array}\right.$$ (10)

### 5.3 Searching for prey

If the whale moves beyond the position where the prey exists, then the whale will abandon the previous moving direction and randomly search for other prey in other directions to avoid falling into a local optimum. This helps avoid local optima and the modelling of the whale searching for prey is as follows:.

$$D^{\prime \prime }=|C\cdot X_{rand}-X(t)|$$ (11)

$$X(t+1)=X_{rand}-A\cdot D^{\prime \prime }$$ (12)

where *X*_*rand*_ is a random whale chosen from the current population; *A* and *C* are described in Eqs 3 and 5.

### 5.4 Population initialization

Like most metaheuristic algorithms, WOA uses pseudo-random numbers for population initialization.

$$X_{i,j}=(ub-lb)\cdot Rand+lb$$ (13)

where *X*_*i*,*j*_ is randomly produced population; *ub* and *lb* are the upper limit and lower limit of the problem; *Rand* is a random number between 0 and 1.

This approach, while simple and direct, often results in poor diversity and uneven distribution of solutions, which can lead to inefficiency in the search process.

### 5.5 Advantages and disadvantages of WOA

The pseudo-code of WOA is shown in Algorithm 1.

It is shown by Algorithm 1 that the structure of WOA is relatively simple, with fewer parameters, making it easy to understand and implement. By dynamically adjusting the control parameter *a*, WOA balances global exploration in the early stages and local exploitation in the later stages, preventing premature convergence to local optima. However, WOA has limitations, particularly in complex multi-modal problems, where it may not explore the search space adequately and may converge prematurely. Moreover, WOA is sensitive to the initial parameter settings, especially the control parameter *a*. Therefore, there is considerable room for improvement in WOA. In the proposed improved WOA (LSWOA), we introduce the Good Nodes Set initialization for generating a more uniform distribution of the population, redesign the logic for Searching for Prey mechanism, and modify the bubble-net attacking phase with new mechanisms DGPS and SEP for balancing exploration and exploitation.

**Algorithm 1 WOA.**

**Begin**

(*Pseodo-random number initialization*)

Initialize the population using Pseodo-random number method;

Initialize the parameters (T,N,p,etc.);

Calculate the fitness of each search agent;

The best search agent is ${\text{X}}^{*}$;

**while** *t*<*T*

**for** each search agent

Update *a*, *A*, *C*, *l*, and *p*;

**if** *p*<0.5

**if** |A|<1

(*Search for prey*)

Update the position of the current search agent by

Eq 2;

**else**

(*Encircling prey*)

Update the position of the current search agent by

Eq 12;

**end if**

**else**

(*Spiral updating*)

Update the position of the current search agent by

Eq 6;

**end if**

**end for**

Check if any search agent goes beyond the search space

and amend it;

Calculate the fitness of each search agent;

Update ${\text{X}}^{*}$ if there is a better solution;

*t* = *t* + 1

**end while**

**return** ${\text{X}}^{*}$

**End**

## 6 LSWOA

### 6.1 Good nodes set initialization

**Fig 2:**
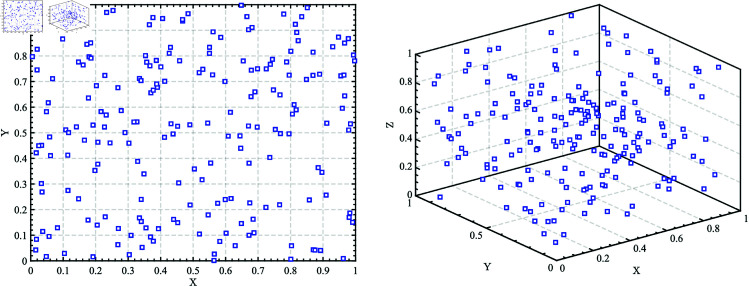
Population initialized by Pseudo-Random Number.

The canonical WOA uses pseudo-random number to generate the population, as shown in Fig 2. While simple and effective, this approach often results in poor population diversity and uneven distribution. This leads to inefficient searches, especially when individuals cluster together.

**Fig 3:**
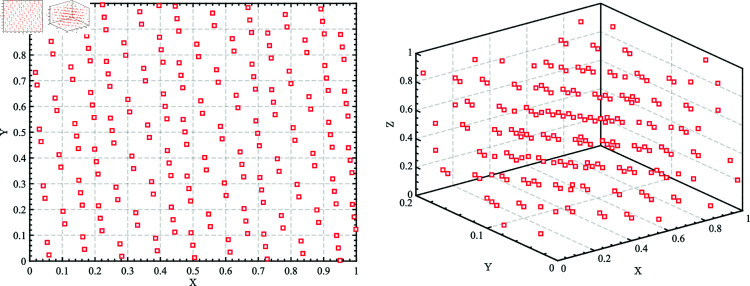
Population initialized by Good Nodes Set.

To address these shortcomings, we adopt the Good Nodes Set (GNS) method for initialization [31], which ensures a more uniform distribution of solutions. The concept of GNS, first introduced by Chinese mathematician Loo-keng Hua, is a method for generating evenly distributed points. This advantage is evident not only in two-dimensional space but also in high-dimensional spaces, as the construction of GNS is dimension-independent. Thus, GNS initialization can enhance the quality of whale populations and improve the exploration capabilities of WOA.The population generated by the Good Nodes Set initialization for a population size of *N*=200 is shown in Fig 3. In comparison to the pseudo-random number method, the population generated by the Good Nodes Set initialization is more uniformly distributed, effectively avoiding the phenomenon of individual clustering. Assuming ${\text{U}}^{\text{D}}$ is a unit hypercube in a *D*-dimensional Euclidean space, the form of the Good Nodes Set can be described by Eq 14:

$$P_{r}^{M}=\{p(k)=(\{kr\},\{kr^{2}\},\ldots ,\{kr^{D}\})|k=1,2,\ldots ,M\}$$ (14)

where {*x*} represents the fractional part of *x*; *M* is the number of points; *r* is a deviation parameter greater than zero; the constant C(r,ε) is associated only with *r* and ε is related to and is a constant greater than zero.

This set $P_{r}^{M}$ is called Good Nodes Set and each node *p*(*k*) in it is called a Good Node. Assume that the upper and lower bounds of the ${\text{i}}^{\text{th}}$ dimension of the search space $x_{max}^{i}$ are and $x_{min}^{i}$, then the mapping formula for mapping the Good Nodes Set to the actual search space is:

$$x_{k}^{i}=x_{min}^{i}+p_{i}(k)\cdot (x_{max}^{i}-x_{min}^{i})$$ (15)

### 6.2 Distance-guided prey searching (DGPS)

As shown in Eqs 11 and 12, in the original WOA, the position update of the whales relies on a randomly selected individual. The current position is updated by moving towards this randomly chosen individual. Due to the dependence on randomly selected individuals, the original WOA tends to favor exploration, ensuring that the algorithm does not converge prematurely. However, this also leads to low exploitation efficiency, poor convergence accuracy, and slower convergence speed, often resulting in the algorithm getting trapped in local optima during later stages. Additionally, the simplicity of this process can lead to overly uniform search behavior in the population, particularly in the later stages, which increases the likelihood of falling into local optima.

To address these issues, this paper proposes a Distance-Guided Prey Searching strategy (DGPS), modeled as follows:

$$D_{1}=|C\cdot X^{*}(t)-X(t)|$$ (16)

$$D_{2}=|X^{*}(t)-X(t)|$$ (17)

$$X(t+1)=X^{*}(t)-A\cdot D_{1}-\omega \cdot D_{2}\cdot Rand$$ (18)

where ${\text{X}}^{*}(\text{t})$ represents the position of the current best solution; *A* and *C* are coefficient vectors, calculated as shown in Eqs 3 and 4; *X* represents the whale’s position; and ω is an inertia weight decreasing from 0.9 to 0, as defined in Eq 27.

DGPS takes into account the distance between the whale’s current position and the current optimal solution and introduces an inertia weight. This strategy emphasizes the dependence on the best solution during position updates. Additionally, randomness and weight adjustments are incorporated into the update process, with the inertia weight ω decreasing from 0.9 to 0, dynamically adjusting the individual’s step size. The stochastic component provides more search directions for the whale, enhancing the algorithm’s exploration capability in the early stages, while allowing for gradual convergence to more accurate solutions later on. This strategy increases the diversity of individual searches, prevents premature convergence of the population, and improves the algorithm’s global search capability.

### 6.3 Spiral encircling prey (SEP)

The encircling prey strategy in the original WOA is relatively simple: individuals move directly towards the leader, with step sizes controlled by the parameter *A*. While this linear update mechanism offers strong exploratory capabilities in the early stages, individual behavior becomes more homogeneous as iterations progress, leading to a loss of exploratory capability in later stages. When trapped in local optima, the WOA lacks effective mechanisms to escape.

**Fig 4:**
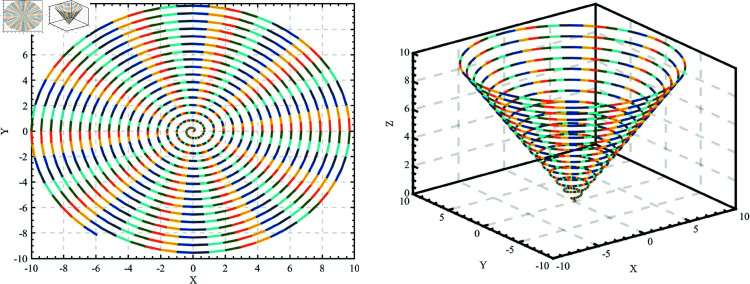
Simulation of Spiral flight.

Inspired by spiral flight, which is shown in Fig 4, this paper introduces a novel Spiral Encircling Prey mechanism (SEP), modeled as follows:

$$X(t+1)=X^{*}(t)+e^{Z\cdot L}\cdot cos(2\pi L)\cdot |A\cdot D|$$ (19)

$$D=|C\cdot X^{*}(t)-X(t)|$$ (20)

L=2·r−1 (21)

$$Z=e^{k\cdot cos(\pi \cdot (1-\frac{t}{T}))}$$ (22)

where ${\text{X}}^{*}(\text{t})$ represents the position of the current best solution; *X* is the whale’s position; *A* and *C* are coefficient vectors, calculated as shown in Eqs 3 and 4; *r* is a random number between 0 and 1; *Z* is the step size of the Spiral flight; *L* and *k* are spiral coefficients.

The proposed spiral encircling prey strategy introduces a nonlinear decay factor *Z*, related to the number of iterations. This factor induces a nonlinear change in the step size as the individual approaches the current optimal solution, enhancing the diversity of search behaviors. In particular, it provides finer-grained searches in the later stages, thus improving convergence accuracy. In the original strategy, the encircling process is direct and linear towards the leader. As the iteration count increases, *A* gradually decreases, causing individuals to converge near the leader. While this promotes rapid convergence, it lacks mechanisms to escape local optima. The spiral encircling prey strategy, by introducing the random variable *L* and a trigonometric oscillation term, introduces stochastic disturbances, ensuring that individuals retain some randomness when approaching the leader. This helps avoid complete convergence to local optima and enhances global search ability. Moreover, by introducing the randomness of Land the non-linearity of *Z*, the spiral encircling prey strategy provides greater flexibility and uncertainty, improving the algorithm’s ability to escape local optima when dealing with complex, multi-modal optimization problems.

### 6.4 Enhanced spiral updating

While the original spiral updating strategy of WOA aids in local search, the lack of randomness and perturbation mechanisms may cause individuals to converge around the leader in a short time, limiting the ability to escape local optima. To address this, this paper proposed an Enhanced Spiral Updating strategy by introducing Levy flight and inertia weight into the spiral updating strategy.

#### 6.4.1 Levy flight.

**Fig 5:**
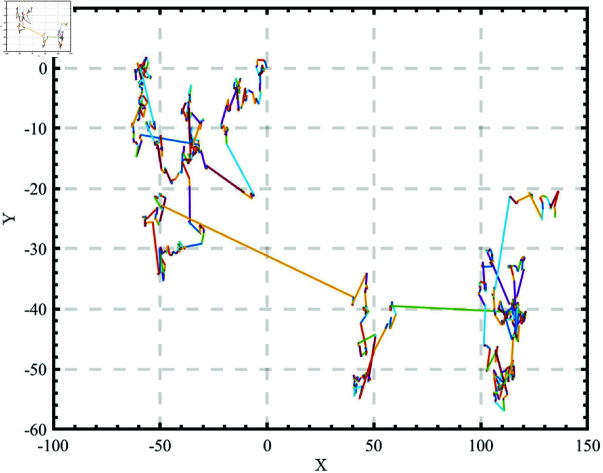
Simulation of Levy flight.

Levy flight is a stochastic walk model based on the Levy distribution, widely used in optimization algorithms, physics, and biology. The concept originates from the work of mathematician Paul Levy in the 1920s. Inspired by foraging behavior observed in nature and jump phenomena in complex systems, Levy flight combines short-range exploration with long jumps, similar to the foraging paths of predators like sharks, birds, and insects. Fig 5 is a simulation of Levy flight. The step size *L*(*s*) of Levy flight follows the Levy distribution, calculated as follows:

$$L(s)=\frac{u}{|\nu {|}^{\frac{1}{\beta }}}$$ (23)

where *u* and ν are normally distributed; β=1.5.

$$u\sim 𝒩(0,\sigma_{u}^{2})$$ (24)

v~𝒩(0,1) (25)

The calculation of $\sigma_{u}$ is given by:

$$\sigma_{u}={\left(\frac{\Gamma (1+\beta )\cdot \sin\left(\frac{\pi \beta }{2}\right)}{\Gamma \left(\frac{1+\beta }{2}\right)\cdot \beta \cdot 2^{\frac{\beta -1}{2}}}\right)}^{\frac{1}{\beta }}$$ (26)

#### 6.4.2 Inertia weight ω.

The concept of inertia weight ω, introduced by Shi *et al*. into the Particle Swarm Optimization (PSO) algorithm [32], significantly enhanced performance by balancing global exploration and local exploitation. Therefore, this paper incorporated an inertia weight ω based on Sigmoid function into the encircling prey strategy of WOA, as shown in Eq 27 [33]. The proposed inertia weight ω increases from 0 to 0.9, starting with a slow increase, followed by a rapid rise in the middle phase, and finally a slow increase towards the end.

$$\omega =\frac{0.9}{1+e^{-k_{1}(\frac{t}{T}-0.5)}}$$ (27)

where *t* is the current iteration; *T* is the maximum number of iterations; *k*_1_ is the slope parameter of the Sigmoid function and the value of the parameter *k*_1_ will be discussed in **Experiment on choosing *k***_**1**_ **and *k***_**2**_.

**Fig 6:**
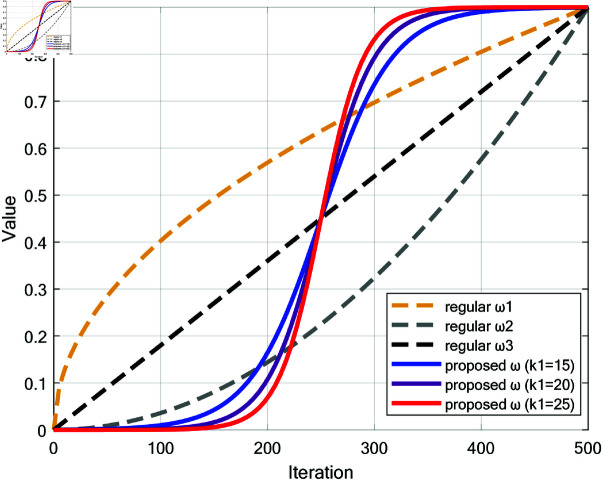
Comparison of different types of inertia weight ω.

During the early stages, a small inertia weight helps reduce excessive jumps, thus enhancing the rationality of global exploration. Despite the lower inertia, the combination of spiral ascent and Levy flight ensures strong global search capabilities, effectively exploring the search space. As iterations progress into the middle phase, the inertia weight increases rapidly, with the algorithm increasingly relying on the current state and historical paths. In the later stages, the inertia weight approaches 0.9, focusing on fine-tuning the optimal solution. This gradual increase in inertia promotes precise convergence while maintaining diversity through Levy flight, avoiding premature convergence. Fig 6 compares the inertia weights proposed in this paper (with *k*_1_=10, 15, 20 respectively) with regular inertia weights $\omega_{1}$, $\omega_{2}$ and $\omega_{3}$.

$$\omega_{1}=0.9\cdot \sqrt{\frac{t}{T}}$$ (28)

$$\omega_{2}=0.9\cdot (\frac{t}{T}{)}^{2}$$ (29)

$$\omega_{3}=0.9\cdot \frac{t}{T}$$ (30)

The Enhanced Spiral Updating strategy, incorporating Levy flight and inertia weight, was modeled as follows:

$$X(t+1)=\omega \cdot X^{*}(t)\cdot L(s)+D^{\prime }\cdot e^{bl}\cdot \cos(2\pi l)$$ (31)

where ${\text{X}}^{*}(\text{t})$ represents the position of the current best solution; ω is the proposed inertia weight; *L*(*s*) is the step size of the Levy flight; *b* is a constant that defines the shape of a logarithmic spiral, usually set to 1; the value of spiral coefficient *l* is [–2,1], and *l* is calculated in Eq 8; *X* is the whale’s position; *a*_1_ is the parameter of the linear change of [–2,–1], calculated in Eq 9; *Rand* is a random number between 0 and 1.

### 6.5 Convergence factor *a*

**Fig 7:**
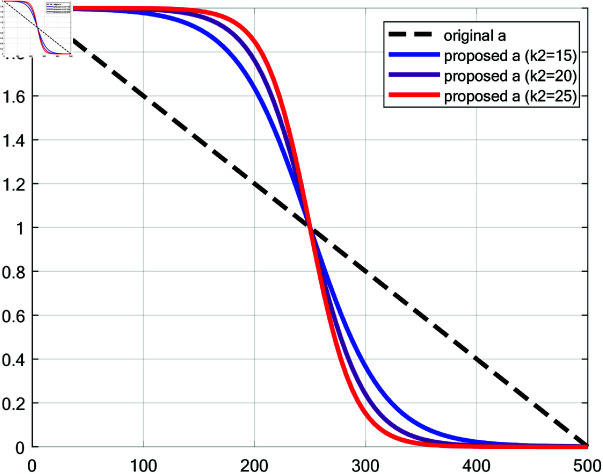
Comparison of the original a and the proposed a.

This paper proposed a convergence factor *a* based on Sigmoid function for the update of coefficient *A* to balance global exploration and local exploitation, as shown in Eq 32. Fig 7 compares the proposed convergence factor *a* (with *k*_2_=15, 20, 25 respectively) with the original linear decreasing one. The proposed update method of convergence factor *a* slows the reduction in the early stage, accelerates it during the middle phase, and slows it again towards the end. This behavior mimics a more complex transition process, providing different convergence characteristics at various stages: slow reduction in the early phase promotes extensive exploration; rapid reduction in the middle phase accelerates convergence; and slow reduction in the later phase preserves some exploration capability while enhancing local exploitation. This strategy provides better balance between global and local search, improving flexibility in convergence behavior, mitigating premature convergence, enhancing adaptability to diverse search environments and improving solution accuracy.

$$a=2-\frac{2}{1+e^{-k_{2}(\frac{t}{T}-0.5)}}$$ (32)

where *t* is the current iteration count; *T* is the maximum number of iterations; *k*_2_ is the slope parameter of the Sigmoid function and the value of the parameter *k*_2_ will be discussed in **Experiment on choosing *k***_**1**_ **and *k***_**2**_.

### 6.6 Time complexity analysis

#### 6.6.1 Time complexity of WOA.

Assume that the time complexity of initialization in WOA is *O*(*ND*). During each iteration, the time complexity of boundary checking is *O*(*ND*), the time complexity of fitness evaluation is *O*(*ND*), and the total time complexity of position updates is *O*(*ND*). Therefore, the total time complexity per iteration is *O*(*ND*). If the algorithm iterates *T* times, the total time complexity is calculated as:

Total Time Complexity1 = Initialization + *T* * (the total time complexity per iteration) = *O*(*ND*) + *T* * *O*(*ND*) = *O*(T*ND).

#### 6.6.2 Time complexity of LSWOA.

Assume that the time complexity of initialization in LSWOA is *O*(*ND*). During each iteration, the time complexity of boundary checking is *O*(*ND*), the time complexity of fitness evaluation is *O*(*ND*), and the total time complexity of position updates is *O*(*ND*). Therefore, the total time complexity per iteration is *O*(*ND*). If the algorithm iterates *T* times, the total time complexity is calc ulated as:

Total Time Complexity2 = Initialization + *T* * (the total time complexity per iteration) =*O*(*ND*) + *T* * *O*(*ND*) = *O*(T*ND)

In summary, the time complexity of LSWOA and WOA are the same, both are *O*(T*ND).

### 6.7 Pseudo-code of LSWOA

The pseudo-code of LSWOA is shown in Algorithm 2

**Algorithm 2 LSWOA.**

**Begin**

(*Good Nodes Set Initialization*)

Initialize the population using Good Nodes Set method;

Initialize the parameters $\left(T,N,p,k_{1},k_{2},\text{etc.}\right)$;

Calculate the fitness of each search agent;

The best search agent is ${\text{X}}^{*}$;

**while** *t*<*T*

**for** each search agent

Update *a*, *A*, *C*, *l*, and *p*;

**if** *p*<0.5

**if** |A|<1

(*Spiral-based Encircling Prey*)

Update the position of the current search agent by

Eq 19;

**else**

(*Distance-Guided Prey Searching*)

Update the position of the current search agent by

Eq 18;

**end if**

**else**

(*Enhanced Spiral Updating*)

Update the position of the current search agent by

Eq 31;

**end if**

**end for**

Check if any search agent goes beyond the search space

and amend it;

Calculate the fitness of each search agent;

Update ${\text{X}}^{*}$ if there is a better solution;

*t* = *t* + 1

**end while**

**return** ${\text{X}}^{*}$

**End**

## 7 Experiments

The experimental setup for this study includes a Windows 11 (64-bit) operating system, an Intel(R) Core(TM) i5-8300H CPU @ 2.30GHz processor, and 8GB of RAM. The simulation platform used is MATLAB R2023a. The algorithm was tested on 23 classical benchmark functions, as shown in Table 10. To verify the performance and effectiveness of LSWOA, the following experiments were conducted.

1. A parameter sensitivity analysis experiment was performed on different LSWOAs with vaious *k*_1_ and *k*_2_, aiming at choosing the perfect option of *k*_1_ and *k*_2_ for convergence factor *a* and inertia weight ω respectively to better balance exploration and exploitation.
2. A qualitative analysis experiment was performed by applying LSWOA on the benchmark functions to comprehensively evaluate the performance, robustness and exploration-exploitation balance of LSWOA in different types of problems, by assessing convergence behavior, population diversity and exploration-exploitation capability.
3. Ablation study was performed by removing each of the five improvement strategies from LSWOA and testing on benchmark functions.
4. LSWOA was compared with the canonical WOA and several SOTA metaheuristic algorithms on the benchmark functions on dimension of 30;
5. LSWOA was compared with the canonical WOA and SOTA algorithms on the benchmark functions on higher dimension of 50 and 100.

### 7.1. Parameter sensitivity analysis experiment

As shown in section 6.4.2 and section 6.5, the inertia weight ω with different *k*_1_ and convergence factor *a* with different *k*_2_ may greatly affect the performance of LSWOA. The ω with different *k*_1_ affects the rate at which the dependence of the whale individuals on the leader changes during position updates in the Enhanced Spiral Updating strategy. Meanwhile, since convergence factor *a* affects the balance of exploration and exploitation, it is necessary to explore the shape of the Sigmoid function of convergence factor *a*.

In this experiment, we discussed the value of *k*_1_ and *k*_2_. To be specific, we execute a Friedman test of LSWOAs with different combination of *k*_1_ (*k*_1_=15, 20, 25) and *k*_2_ (*k*_2_=15, 20, 25) on the benchmark functions. The number of iterations was set to *T*=500, and the population size to *N*=30. Each LSWOA with different *k*_1_ and *k*_2_ was executed 30 times on the 23 benchmark functions listed in Table 10. And the Friedman values were recorded in Table 2. The results shown that the LSWOA with *k*_1_=20 and *k*_2_=25 performed the best. Experimental results show that the LSWOAs with *k*_2_=25 generally perform well. However, when *k*_2_ = 25, the LSWOA with (*k*_1_=25, *k*_2_=25) and the LSWOA with (*k*_1_=15, *k*_2_=25) did not perform as well as the LSWOA with (*k*_1_=20, *k*_2_=25). In complex functions, a Sigmoid function with a steeper change in the middle (larger *k*_1_) or a Sigmoid function with a more gradual change in the middle (smaller *k*_1_) does not necessarily help LSWOA escape from local optima more effectively. Therefore, *k*_1_ is not always better when it is larger. Therefore, we chose *k*_1_=20 and *k*_2_=25.

**Table 2:** Results of parameter sensitivity analysis experiment. *LSWOA*(20,25) means *k*_1_=20, *k*_2_=25; *LSWOA*(25,20) means *k*_1_=25, *k*_2_=20.

Function	LSWOA(15,15)	LSWOA(15,20)	LSWOA(15,25)	LSWOA(20,15)	LSWOA(20,20)	LSWOA(20,25)	LSWOA(25,15)	LSWOA(25,20)	LSWOA(25,25)
F1	5.0000	5.0000	5.0000	5.0000	5.0000	5.0000	5.0000	5.0000	5.0000
F2	5.0000	5.0000	5.0000	5.0000	5.0000	5.0000	5.0000	5.0000	5.0000
F3	5.0000	5.0000	5.0000	5.0000	5.0000	5.0000	5.0000	5.0000	5.0000
F4	5.0000	5.0000	5.0000	5.0000	5.0000	5.0000	5.0000	5.0000	5.0000
F5	7.9333	4.9000	2.5667	7.6333	4.7000	2.0667	7.6333	5.3667	2.2000
F6	7.4667	4.9000	2.1333	7.7000	4.9333	2.1000	7.9333	4.9000	2.9333
F7	5.2000	5.7667	4.9000	5.0333	5.3000	4.8333	4.3000	4.9000	4.7667
F8	8.2333	4.7667	2.0333	7.4333	4.9667	1.9333	8.0000	5.4000	2.2333
F9	5.0000	5.0000	5.0000	5.0000	5.0000	5.0000	5.0000	5.0000	5.0000
F10	5.0000	5.0000	5.0000	5.0000	5.0000	5.0000	5.0000	5.0000	5.0000
F11	5.0000	5.0000	5.0000	5.0000	5.0000	5.0000	5.0000	5.0000	5.0000
F12	7.1333	3.5667	5.9000	6.7667	3.7000	3.3000	6.5333	3.4333	4.6667
F13	7.0667	3.9333	4.2667	7.0333	4.0000	3.3333	6.7333	4.2667	4.3667
F14	7.3333	4.7833	2.4667	7.7667	5.1500	2.1500	7.8000	5.3000	2.8667
F15	4.1000	4.8667	5.6333	4.9333	5.4000	5.5333	5.0333	4.5333	4.9667
F16	7.7000	5.3333	2.2000	7.3333	5.1000	2.4667	8.0000	4.6333	2.2333
F17	7.8333	4.7667	2.4000	7.7333	5.1000	2.1667	7.9333	4.8000	2.2667
F18	4.5333	5.3000	4.9333	4.0333	4.8000	5.1333	4.4333	5.7000	6.1333
F19	6.0000	4.8000	4.5333	4.7667	4.3333	5.5667	3.7000	5.4000	5.9000
F20	7.4667	5.1333	2.8000	7.2333	4.8667	2.6333	7.1333	4.9333	2.8000
F21	8.2667	4.9000	2.1333	8.0667	4.9333	2.1000	7.5333	5.2667	1.8000
F22	8.1333	4.9000	2.1333	7.9333	5.1667	2.0333	7.8333	5.0000	1.8667
F23	7.8667	4.9667	2.0667	7.9333	5.0667	2.2000	8.1333	4.9667	1.8000
Average	6.4029	4.8949	3.8304	6.2754	4.8920	3.6761	6.2464	4.9478	3.8609
Rank	9	5	2	8	4	1	7	6	3

### 7.2. Qualitative analysis experiment

**Fig 8:**
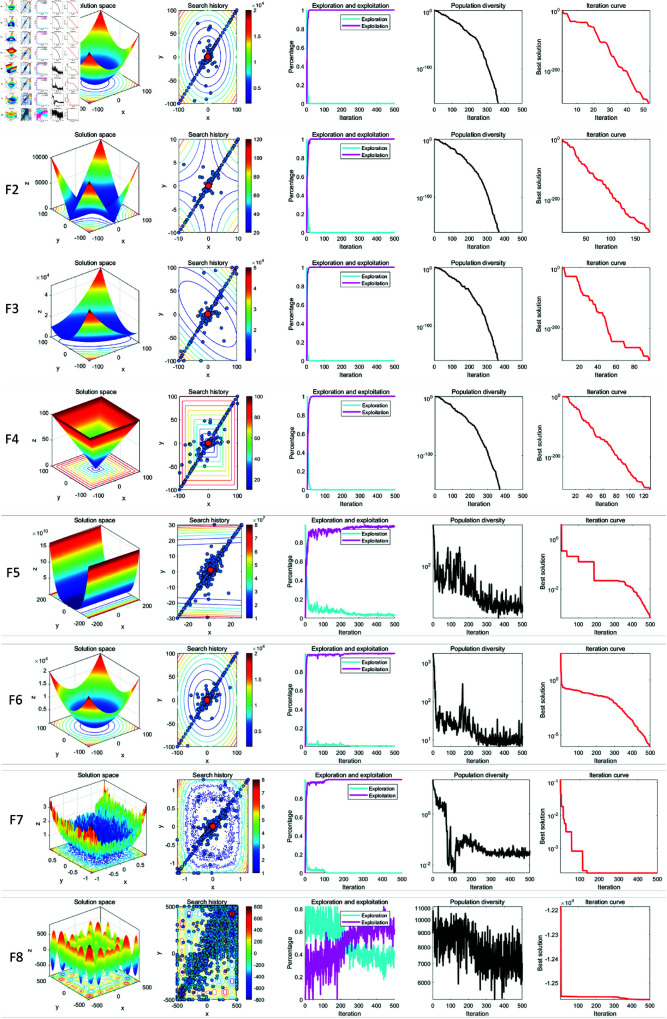
Performance of LSWOA in qualitative analysis experiment (F1-F8).

**Fig 9:**
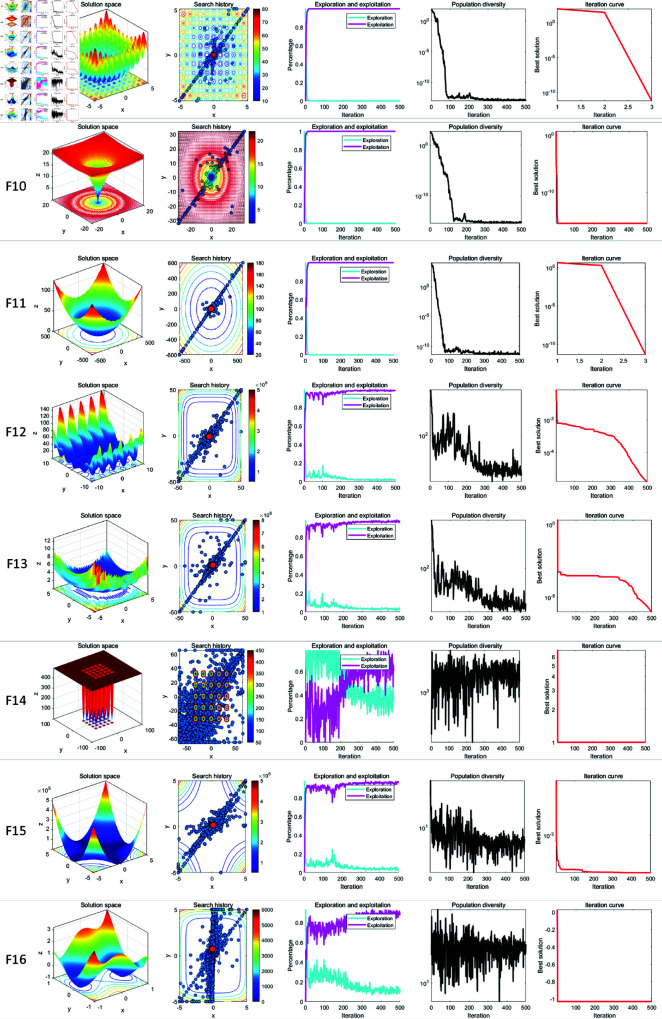
Performance of LSWOA in qualitative analysis experiment (F9-F16).

**Fig 10:**
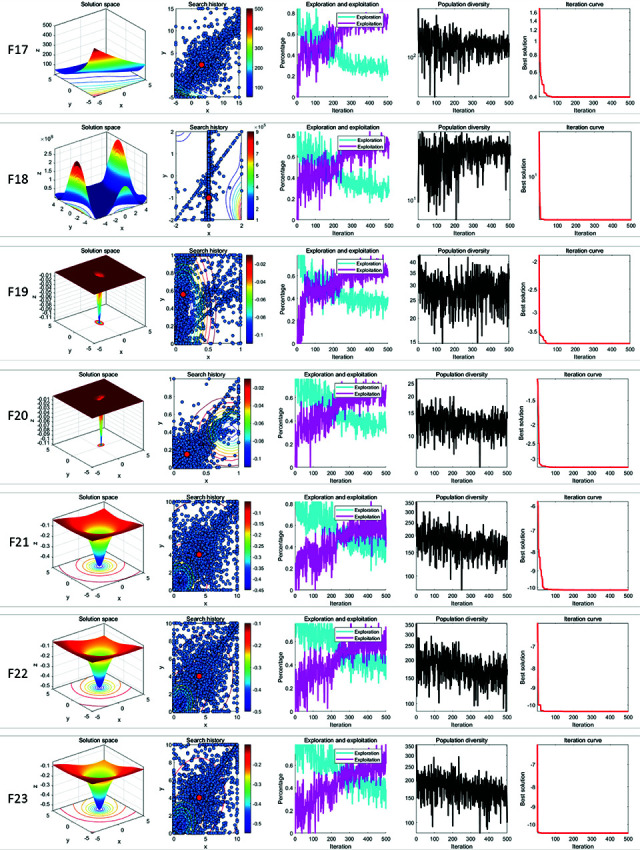
Performance of LSWOA in qualitative analysis experiment (F17-F23).

In qualitative analysis experiment, we applied LSWOA on the benchmark functions recorded the search history of the whale individuals, the exploration-exploitation percentage of LSWOA during the iterations and the polulation diversity of LSWOA. So that we could comprehensively evaluate the performance, robustness and exploration-exploitation balance of LSWOA in different types of problems. In this experiment, the maximum number of iterations was set to *T*=500 and the population size was *N*=30. The search history of whale individuals, the proportions of exploration and exploitation, population diversity, and iteration curves were recorded and are presented in Figs 8–10, which includes:

- the landscape of benchmark functions;
- the search history of the whale population;
- the exploration-exploitation ratio;
- the changes in population diversity;
- the iteration curves.

It is noteworthy that, from the figures, it is evident that the whale individuals in LSWOA demonstrate a well-distributed search within the solution space, indicating the effectiveness of the Good Nodes Set initialization method. For the F8, the global optimal solution is located in the upper-right corner of the solution space, posing significant challenges for LSWOA’s ability to escape local optima. As shown in F17-F23, the Distance-Guided Prey Searching strategy enabled elite whales to guide the remaining whales towards the global optimal region, avoiding ineffective exploration of the whales. The oscillatory component introduced in the Spiral Encircling Prey strategy facilitates detailed exploration around the elite whales, ultimately leading to the identification of the optimal solution for F8.

Furthermore, the introduction of Levy flight in the Enhanced Spiral Updating strategy, combined with the oscillatory component in the Spiral Encircling Prey strategy, allows LSWOA to consistently escape local optima and maintain high population diversity, even when addressing complex combinatorial problems such as F15-F23.

In terms of balancing exploration and exploitation, LSWOA performs excellently. For uni-modal functions (F1-F7), the results show that the exploitation proportion of LSWOA increases rapidly during the iterative process, demonstrating strong exploitation capabilities. For compositional functions (F16-F23), the exploration proportion decreases gradually in the early iterations, reflecting LSWOA’s robust global exploration ability. In the later stages of the iterations, the exploitation proportion increases significantly, indicating strong local exploitation capabilities. Furthermore, the exploration-exploitation proportion curves highlight the smooth transition between exploration and exploitation achieved through the update method of convergence factor *a* based on Sigmoid function.

### 7.3. Ablation study

In the ablation study, each of the five improvement strategies was removed from LSWOA:

1. LSWOA1: The LSWOA where Good Nodes Set initialization was replaced with pseudo-random number initialization.
2. LSWOA2: The LSWOA where the Distance-guided prey searching strategy was replaced with the prey searching strategy of the original WOA.
3. LSWOA3: The LSWOA where the Spiral Encircling Prey strategy was replaced with the encircling prey strategy of the original WOA.

- LSWOA4: The LSWOA where the Enhanced Spiral Updating strategy was replaced with the Spiral Updating strategy of the original WOA.
- LSWOA5: The LSWOA where the proposed update method of the convergence factor *a* was replaced with the update method in the original WOA.

**Fig 11:**
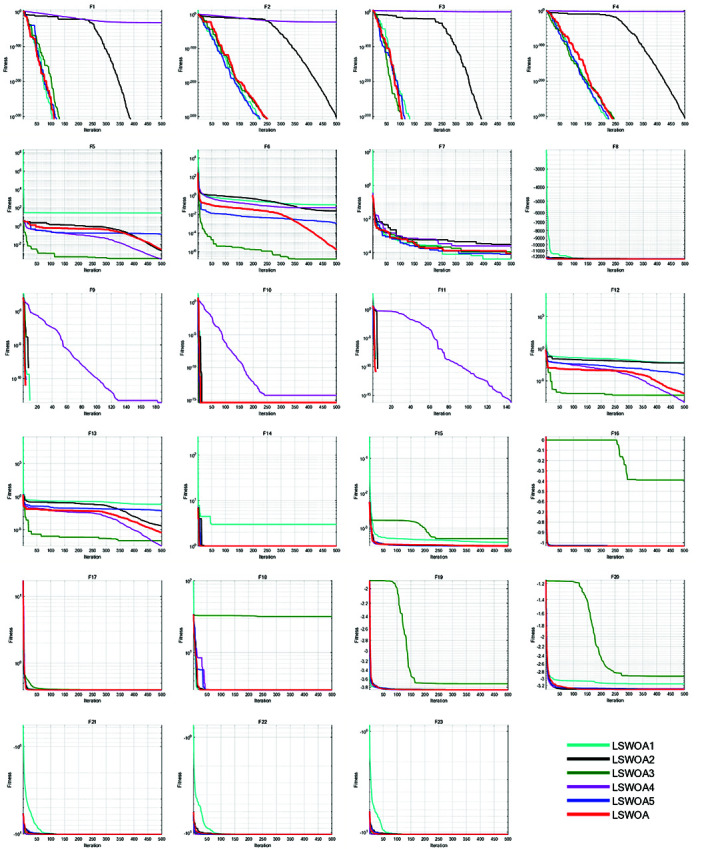
Iterative curves of the algorithms in ablation study.

The number of iterations was set to *T*=500, and the population size to *N*=30. Each algorithm was executed 30 times on the 23 classical benchmark functions listed in Table 10. Performance analysis was conducted, and the iterative curves are shown in Fig 11.

From Fig 11, it can be observed that the Good Nodes Set initialization provides a uniformly distributed population of whales, allowing for better handling of problems such as F5 and F12. The uniform distribution helps WOA explore the search space more efficiently, improving both speed and accuracy in solving problems like F8, F20-F23. The distance-guided prey search strategy, by considering the whale’s current position relative to the best-known solution, increases dependency on the optimal solution during position updates. The introduced random component adds diversity to search directions, enhancing early exploration capability, leading to faster convergence in solving uni-modal problems. The spiral encirclement strategy introduces random perturbations, strengthening the global search ability, which improves the handling of complex problems like F15-F16 and F18-F20. The improved prey capture strategy integrates Levy Flight and inertia weight concepts. The combination of Levy Flight and inertia weights based on Sigmoid function enhances the dynamic balance between exploration and exploitation, significantly improving accuracy and speed when solving problems such as F1-F4. In the later stages, this allows the algorithm to focus on local exploitation, giving it an edge in addressing F9-F11. The coefficient a based on Sigmoid function provides a better balance between global and local search, enhancing solution accuracy and early convergence while improving adaptability to varying search environments. This allows the algorithm to better handle complex optimization problems like F5-F6 and F12-F13.

### 7.4. Comparison experiment with different metaheuristic algorithms

**Fig 12:**
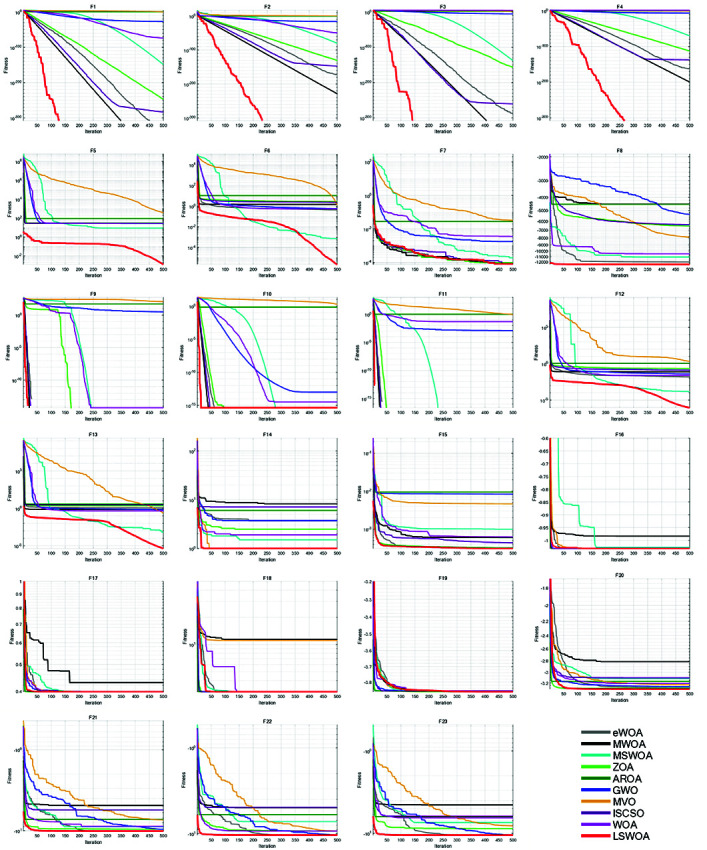
Iterative curves of the algorithms in comparison experiment.

To further verify the effectiveness of LSWOA, eWOA [34], MWOA [35], MSWOA [36], Zebra Optimization Algorithm (ZOA) [37], Attraction-Repulsion Optimization Algorithm (AROA) [38], Grey Wolf Optimizer (GWO) [39], Multi-Verse Optimizer (MVO) [40], ISCSO [41] and Whale Optimization Algorithm (WOA) were selected for comparison. These algorithms were tested on 23 classical benchmark functions listed in Table 10 [42]. Details of the algorithms were shown in Table 4. The parameter settings for each algorithm were shown in Table 3. The number of iterations was set to *T*=500, and the population size to *N*=30. Each algorithm was run independently 30 times on the 23 benchmark functions, with the average fitness (Ave), standard deviation (Std), *p*-value from Wilcoxon rank-sum test, and Friedman values recorded for parametric and non-parametric analysis. The experimental results were shown in Fig 12 and Table 5.

**Table 3:** Parameter settings for metaheuristic algorithms.

Algorithm	Parameter	Value
eWOA [34]	Convergence Factor *a*	2 decrease to 0
	Spiral Factor *b*	1
	Weight Vector ω	0.3 increasing to 0.6
	Selection Parameter β	1 decreasing to 0
MWOA [35]	Convergence Factor *a*	2 decreasing to 0
	Spiral Factor *b*	1
	*CF*_1_	2.5
	*CF*_2_	2.5
MSWOA [36]	Convergence Factor *a*	2 decreasing to 0
	Spiral Factor *b*	1
	Inertia Weight ω	From 0.5 to 4.5
ZOA [37]	*R*	0.1
AROA [38]	Attraction factor *c*	0.95
	Local search scaling factor 1	0.15
	Local search scaling factor 2	0.6
	Attraction probability 1	0.2
	Local search probability	0.8
	Expansion factor	0.4
	Local search threshold 1	0.9
	Local search threshold 2	0.85
	Local search threshold 3	0.9
GWO [39]	Convergence Factor *a*	2 decreasing to 0
MVO [40]	*WEP*_*max*_	1
	*WEP*_*min*_	0.2
ISCSO [41]	Maximum Sensitivity Range *S*	2
	*R*	[-2, 2]
WOA [30]	Convergence Factor *a*	2 decrease to 0
	Spiral Factor *b*	1
LSWOA	Convergence Factor *a*	2 decrease to 0
	Spiral Factor *b*	1
	Inertia weight ω	0 increase to 0.9
	Spiral Factor *k*	1
	*k*_1_	20
	*k*_2_	25

**Table 4:** Details of the metaheuristic algorithms in comparison experiments.

Algorithm	Year	Author(s)	Source of Inspiration
eWOA [34]	2021	Chakraborty S *et al*.	A nonlinear weight vector ω and
			a new selection parameter β.
MWOA [35]	2021	Anitha J *et al*.	The cosine function and
			the correction factors.
MSWOA [36]	2022	W Yang *et al*.	Adaptive inertia weight, dynamic
			convergence factor and Levy flight.
Zebra Optimization Algorithm	2022	E Trojovská *et al*.	Foraging and Defense Strategy
(ZOA) [37]			of zebras.
Attraction-Repulsion Optimization	2024	K Cymerys	Attraction-repulsion phenomenon.
Algorithm (AROA) [38]			
Grey Wolf Optimizer (GWO) [39]	2014	S Mirjalili *et al*.	The leadership hierarchy and
			hunting system of gray wolves.
Multi-Verse Optimizer (MVO) [40]	2014	S Mirjalili *et al*.	Concepts in cosmology: white
			hole, black hole, and wormhole.
Improved Sand Cat Swarm	2024	Ying Li *et al*.	Nonlinear convergence strategy,
Optimization algorithm (ISCSO) [41]			perturbation factor
Whale Optimization Algorithm	2016	S Mirjalili *et al*.	The hunting behavior of
(WOA) [30]			humpback whales.

**Table 5:** Results of parametric tests of different algorithms. *Ave* indicates average fitness, *Std* indicates standard deviation.

Function	Metrics	eWOA	MWOA	MSWOA	ZOA	AROA	GWO	MVO	ISCSO	WOA	LSWOA
F1	Ave	0.0000E+00	0.0000E+00	3.0553E-149	5.5860E-249	5.3893E+00	1.2036E-27	1.2290E+00	1.1359E-283	4.6869E-72	0.0000E+00
	Std	0.0000E+00	0.0000E+00	1.0769E-148	7.3421E-249	5.9368E+00	2.3510E-27	4.0059E-01	4.5240E-283	2.5553E-71	0.0000E+00
F2	Ave	1.1002E-172	1.2500E-229	5.9377E-81	2.5007E-131	5.9639E-01	1.0114E-16	3.1416E+00	6.7549E-149	8.0468E-51	0.0000E+00
	Std	4.2342E-172	4.7640E-229	1.4341E-80	7.4471E-131	1.8736E-01	8.8832E-17	1.2104E+01	2.2018E-148	2.5434E-50	0.0000E+00
F3	Ave	1.5358E-285	0.0000E+00	1.8721E-138	1.1929E-153	1.3210E+02	4.9151E-06	2.2398E+02	5.6041E-262	4.2390E+04	0.0000E+00
	Std	4.5636E-285	0.0000E+00	5.1672E-138	6.5340E-153	1.1124E+02	1.1204E-05	1.0270E+02	7.6430E-262	1.4099E+04	0.0000E+00
F4	Ave	4.6723E-166	9.3763E-201	8.5123E-71	1.2073E-113	1.5350E+00	8.6787E-07	2.0568E+00	9.8246E-138	5.1152E+01	0.0000E+00
	Std	6.6244E-166	2.5320E-200	7.3676E-71	6.3858E-113	7.3806E-01	5.4387E-07	9.5222E-01	3.4383E-137	3.1125E+01	0.0000E+00
F5	Ave	2.8425E+01	2.8691E+01	4.8491E+00	2.8476E+01	8.7544E+01	2.7209E+01	4.0931E+02	2.7770E+01	2.8080E+01	1.8308E-03
	Std	2.6860E-01	1.2488E-01	1.0739E+01	3.5952E-01	5.3097E+01	8.0151E-01	6.9345E+02	8.5619E-01	4.9696E-01	2.4230E-03
F6	Ave	3.0566E-01	1.3074E+00	1.0784E-03	2.7042E+00	1.1493E+01	7.9813E-01	1.2620E+00	2.0534E+00	3.9719E-01	2.6657E-06
	Std	1.7444E-01	3.6906E-01	1.3607E-03	5.9314E-01	4.4252E+00	4.8444E-01	3.1719E-01	4.8823E-01	2.0891E-01	9.4722E-06
F7	Ave	1.2810E-04	9.8656E-05	1.5095E-04	9.3895E-05	2.5548E-02	2.3862E-03	3.2376E-02	1.3450E-04	3.5346E-03	9.0776E-05
	Std	8.9796E-05	1.0068E-04	1.1470E-04	7.3981E-05	1.7413E-02	1.1196E-03	1.4796E-02	1.4321E-04	5.2991E-03	6.8272E-05
F8	Ave	-1.1803E+04	-4.6999E+03	-9.4453E+03	-6.6240E+03	-4.6470E+03	-6.1760E+03	-7.4646E+03	-6.7086E+03	-1.0001E+04	-1.2569E+04
	Std	9.0408E+02	1.5840E+03	1.3596E+03	6.1647E+02	6.9661E+02	8.3235E+02	6.5841E+02	8.7809E+02	1.6451E+03	7.8694E-03
F9	Ave	0.0000E+00	0.0000E+00	8.7419E-02	0.0000E+00	7.1160E+01	2.6742E+00	1.3469E+02	0.0000E+00	0.0000E+00	0.0000E+00
	Std	0.0000E+00	0.0000E+00	4.7881E-01	0.0000E+00	7.2555E+01	4.2751E+00	3.2261E+01	0.0000E+00	0.0000E+00	0.0000E+00
F10	Ave	4.4409E-16	4.4409E-16	4.4409E-16	4.4409E-16	9.4661E-01	1.0655E-13	1.8241E+00	4.4409E-16	3.2863E-15	4.4409E-16
	Std	0.0000E+00	0.0000E+00	0.0000E+00	0.0000E+00	3.9386E-01	1.6787E-14	5.3817E-01	0.0000E+00	2.5380E-15	0.0000E+00
F11	Ave	0.0000E+00	0.0000E+00	0.0000E+00	0.0000E+00	9.8196E-01	5.7417E-03	8.3080E-01	0.0000E+00	0.0000E+00	0.0000E+00
	Std	0.0000E+00	0.0000E+00	0.0000E+00	0.0000E+00	1.3525E-01	8.0431E-03	8.9282E-02	0.0000E+00	0.0000E+00	0.0000E+00
F12	Ave	1.7775E-02	9.3088E-02	4.6275E-03	1.7441E-01	1.3071E+00	4.3293E-02	2.0070E+00	1.1748E-01	3.1826E-02	1.9474E-04
	Std	7.8288E-03	5.2264E-02	2.3960E-02	7.1389E-02	2.9971E-01	1.9453E-02	1.3266E+00	3.9770E-02	4.8453E-02	5.9581E-04
F13	Ave	5.4798E-01	6.6591E-01	1.2927E-02	2.2756E+00	4.0330E+00	7.0670E-01	1.8812E-01	2.9010E+00	5.3470E-01	7.7536E-03
	Std	6.8072E-01	1.8894E-01	2.0496E-02	3.0511E-01	6.8776E-01	2.9248E-01	9.3055E-02	1.0932E-01	2.8896E-01	3.1116E-03
F14	Ave	2.8386E+00	6.7126E+00	1.9208E+00	2.3805E+00	5.2835E+00	3.3173E+00	9.9800E-01	6.3970E+00	2.3093E+00	9.9800E-01
	Std	2.4455E+00	4.3818E+00	1.6776E+00	2.0284E+00	3.8073E+00	3.6890E+00	4.3405E-11	5.1482E+00	2.5859E+00	4.5773E-14
F15	Ave	3.7141E-04	5.4305E-04	1.1733E-03	1.0448E-03	6.2513E-03	6.4556E-03	3.3293E-03	3.7495E-04	7.6769E-04	3.0859E-04
	Std	2.3124E-04	1.5487E-04	7.5100E-04	3.6560E-03	8.8533E-03	9.2640E-03	6.7973E-03	2.4047E-04	4.9272E-04	1.9233E-06
F16	Ave	-1.0316E+00	-9.6465E-01	-1.0315E+00	-1.0316E+00	-1.0316E+00	-1.0316E+00	-1.0316E+00	-1.0316E+00	-1.0316E+00	-1.0316E+00
	Std	9.6284E-16	8.6697E-02	4.7445E-04	2.2267E-10	1.3186E-04	2.4454E-08	3.7769E-07	8.6580E-09	7.5393E-10	6.3611E-16
F17	Ave	3.9789E-01	4.1225E-01	3.9831E-01	3.9789E-01	3.9982E-01	3.9789E-01	3.9789E-01	3.9789E-01	3.9789E-01	3.9789E-01
	Std	5.5610E-09	1.5990E-02	7.7484E-04	2.4535E-08	6.0517E-03	3.1378E-06	4.5035E-07	1.5506E-06	3.4463E-05	4.7265E-14
F19	Ave	-3.8628E+00	-3.7630E+00	-3.8610E+00	-3.8623E+00	-3.8584E+00	-3.8621E+00	-3.8628E+00	-3.8616E+00	-3.8589E+00	-3.8628E+00
	Std	2.9937E-11	7.4894E-02	1.5137E-03	6.0568E-04	1.0715E-02	1.8345E-03	1.9139E-06	2.6692E-03	5.2981E-03	2.7101E-05
F20	Ave	-3.2682E+00	-2.9017E+00	-3.1097E+00	-3.3088E+00	-3.2110E+00	-3.2599E+00	-3.2614E+00	-3.1702E+00	-3.2160E+00	-3.3180E+00
	Std	7.6082E-02	1.8461E-01	3.1819E-02	3.9947E-02	8.3447E-02	7.7033E-02	6.1622E-02	1.1809E-01	1.0090E-01	2.1707E-02
F21	Ave	-1.0153E+01	-4.8345E+00	-8.0463E+00	-9.8126E+00	-6.1516E+00	-9.3943E+00	-7.2938E+00	-5.3950E+00	-7.7420E+00	-1.0153E+01
	Std	3.8597E-04	1.2790E+00	2.4883E+00	1.2932E+00	3.3244E+00	2.0036E+00	2.7853E+00	1.2932E+00	2.6535E+00	7.3204E-11
F22	Ave	-1.0403E+01	-4.7493E+00	-6.5249E+00	-8.9855E+00	-6.1565E+00	-1.0224E+01	-8.4042E+00	-5.2648E+00	-7.6630E+00	-1.0403E+01
	Std	1.6811E-03	8.3620E-01	3.4795E+00	2.3907E+00	3.2366E+00	9.7018E-01	2.9429E+00	9.7017E-01	3.0167E+00	1.2362E-10
F23	Ave	-1.0536E+01	-4.5665E+00	-6.5492E+00	-9.9955E+00	-5.6277E+00	-1.0264E+01	-7.9666E+00	-5.6691E+00	-7.0320E+00	-1.0536E+01
	Std	9.3600E-04	1.2299E+00	3.6023E+00	1.6501E+00	3.5307E+00	1.4812E+00	3.2985E+00	1.6497E+00	3.2027E+00	5.0191E-11

#### 7.4.1 Parametric analysis.

The results shows that LSWOA outperformed all algorithms in most functions. Compared to the original WOA, the overall performance of LSWOA was significantly improved. From Fig 12 and Table 5, it is evident that, except for F19, LSWOA achieved the best performance in terms of both the average and standard deviation across all algorithms, demonstrating the superior adaptability and robustness of WOA in solving various types of problems. For F1-F4 and F9-F11, LSWOA exhibited a rapid convergence rate and high optimization precision. In F5-F6 and F12-F13, the Spiral Encircling Prey strategy enabled LSWOA to consistently escape local optima. In F14, although both MVO and LSWOA achieved an average fitness of 0.998, LSWOA demonstrated superior stability. In

F16-F20, while the convergence rate of LSWOA was not the fastest, its optimization precision and stability far exceeded the other algorithms. Thanks to the Good Nodes Set Initialization, the uniformly distributed population allows for rapid convergence, high stability, and precision when handling composite problems such as F21-F23. In F19, although LSWOA found the optimal solution, its optimization accuracy and stability were inferior to eWOA and MVO. This supports the ’No Free Lunch’ theorem: LSWOA is not perfect. While LSWOA performed admirably, it may underperform certain algorithms in specific scenarios.

#### 7.4.2 Non-parametric Wilcoxon rank-sum test.

The Wilcoxon rank-sum test, a non-parametric test used to assess whether two independent samples differ significantly in distribution, showed significant differences between LSWOA and GWO across all functions. For F1, there was no significant difference between LSWOA, eWOA and MWOA, as both quickly converged to the optimal solution in 30 tests. For F3, there was no significant difference between LSWOA and MWOA, as both quickly converged to the optimal solution. LSWOA shows no significant difference from eWOA, MWOA, ZOA, ISCSO, and WOA on F9, as they all exhibit faster convergence speeds. Similarly, no significant difference was observed between LSWOA and eWOA, MWOA, MSWOA, ZOA, and ISCSO for F10, as the algorithms rapidly converged to near-optimal solutions in 30 tests. For F11, no significant difference was observed between LSWOA and eWOA, MWOA, MSWOA, ZOA, ISCSO, and WOA, as all algorithms converged quickly.

#### 7.4.3 Non-parametric Friedman test.

The Friedman test, which identifies whether multiple algorithms differ statistically, provides a fair comparison by eliminating sample bias. Based on the average Friedman rank, LSWOA ranked first with an average Friedman value of 1.6543, followed by eWOA in second place. ZOA, ISCSO, MSWOA ranked third, fourth, fifth respectively. WOA, GWO, MWOA ranked sixth, seventh, eighth respectively with MVO in ninth, and AROA in tenth place.

In summary, LSWOA performed exceptionally well across the 23 benchmark functions in dimension of 30, with significant differences compared to the other SOTA algorithms.

### 7.5 Scalability comparison experiment with different metaheuristic algorithms

In the 23 classic benchmark functions, F1-F13 are functions with expandable dimensions, while F14-F23 are functions with fixed dimensions. To validate the ability of LSWOA to handle problems of different dimensions and complexities, this experiment expands the dimensions of the expandable functions (F1-F13) to 50 and 100 dimensions, while keeping the dimensions of the functions (F14-F23) fixed. LSWOA was compared with eWOA, MWOA, MSWOA, ZOA, AROA, GWO, MVO, ISCSO and WOA on the benchmark functions on higher dimension of 50 (*D*=50) and 100 (*D*=100). The parameter settings for each algorithm were shown in Table 3. The number of iterations was set to *T*=500, and the population size was set to *N*=30. Each algorithm was run independently 30 times on the benchmark functions, with the *p*-value from Wilcoxon rank-sum test and Friedman values recorded for performance analysis. The experimental results were shown in Table 7.

**Table 6:** Results of non-parametric tests of different algorithms.

Algorithm	Rank	Average Friedman Value	+/=/-
eWOA	2	3.2275	19/4/0
MWOA	8	6.4732	18/5/0
MSWOA	5	5.6362	21/2/0
ZOA	3	4.7447	20/3/0
AROA	10	8.6768	23/0/0
GWO	7	6.0935	23/0/0
MVO	9	7.0145	23/0/0
ISCSO	4	5.4514	20/3/0
WOA	6	6.0275	21/2/0
LSWOA	1	1.6543	-

**Table 7:** Results of non-parametric tests of different algorithms in higher dimensions.

Dimension	Algorithm	Rank	Average Friedman Value	+/=/-
*D* = 50	eWOA	2	3.2254	18/4/1
	MWOA	8	6.2870	17/5/1
	MSWOA	5	5.6232	21/2/0
	ZOA	3	4.7413	20/3/0
	AROA	10	8.5826	23/0/0
	GWO	7	6.0928	23/0/0
	MVO	9	7.2594	22/1/0
	ISCSO	4	5.5362	19/3/1
	WOA	6	5.8725	22/1/0
	LSWOA	1	1.7797	-
*D* = 100	eWOA	2	3.0652	18/4/1
	MWOA	7	6.0587	17/5/1
	MSWOA	5	5.4862	21/2/0
	ZOA	3	4.8015	20/3/0
	AROA	10	8.4464	23/0/0
	GWO	8	6.3652	23/0/0
	MVO	9	7.6464	22/1/0
	ISCSO	4	5.4268	19/3/1
	WOA	6	5.9783	22/1/0
	LSWOA	1	1.7254	-

The results shown that LSWOA performed excellently in the scalability comparison experiment.

As shown in Table 7, in the experiments with dimensions of 50 and 100, LSWOA achieved the first in the Friedman ranking. Moreover, in the Wilcoxon rank-sum test, there was a significant difference between LSWOA and other SOTA algorithms with the dimensions of 50 and 100. This was sufficient evidence to demonstrate that LSWOA still possessed strong optimization capability when handling optimization problems of different dimensions, and it shown a strong competitive edge compared to other excellent basic metaheuristic algorithms.

Table 8 summarized all performance results of LSWOA and other algorithms by a useful metric named overall effectiveness (OE). In Table 8, *w* indicates win, *t* indicates tie and *l* indicates loss. The OE of each algorithm was computed by Eq 33 [43].

**Table 8:** Effectiveness of LSWOA and other SOTA algorithms with *D*=30, 50 and 100.

Metrics	eWOA	MWOA	MSWOA	ZOA	AROA	GWO	MVO	ISCSO	WOA	LSWOA
	(*w*/*t*/*l*)	(*w*/*t*/*l*)	(*w*/*t*/*l*)	(*w*/*t*/*l*)	(*w*/*t*/*l*)	(*w*/*t*/*l*)	(*w*/*t*/*l*)	(*w*/*t*/*l*)	(*w*/*t*/*l*)	(*w*/*t*/*l*)
*D*=30	0/0/23	0/0/23	0/0/23	0/0/23	0/0/23	0/3/20	0/3/20	0/5/18	0/2/21	18/5/0
*D*=50	1/4/18	1/5/17	1/3/19	1/2/20	0/0/23	0/0/23	0/1/22	0/2/21	0/2/21	17/5/1
*D*=100	1/4/18	1/5/17	0/2/21	0/3/20	0/0/23	0/0/23	0/1/22	1/3/19	0/1/22	17/5/1
Total	2/8/59	2/10/57	1/5/63	1/5/63	0/0/69	0/3/66	0/5/64	1/10/58	0/5/64	52/15/2
*OE*	14.49%	17.39%	8.70%	8.70%	0.00%	4.35%	7.25%	15.94%	7.25%	97.10%

$$OE=\frac{N-L}{L}\cdot 100$$ (33)

where *N* is the total number of tests; *L* is the total number of losing tests for each algorithm.

Resultd proves that LSWOA with the *OE*=97.10%, was competitive with other algorithms on the benchmark functions with various dimensions of *D*=30, *D*=50 and *D*=100. LSWOA was the most effective algorithm among the competitors.

## 8 Engineering design optimization

Compared to canonical engineering design methods, metaheuristic algorithms have been widely applied in engineering design due to their ability to solve complex, multi-objective and constrained problems. In this section, seven engineering design problems were used to test the performance of LSWOA to test the effectiveness and applicability of LSWOA.

**Fig 13:**
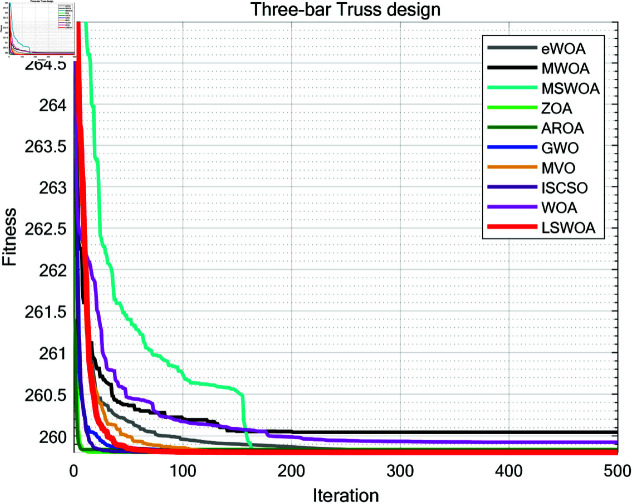
Iteration curves of the algorithms in the Three-bar Truss design problem.

**Fig 14:**
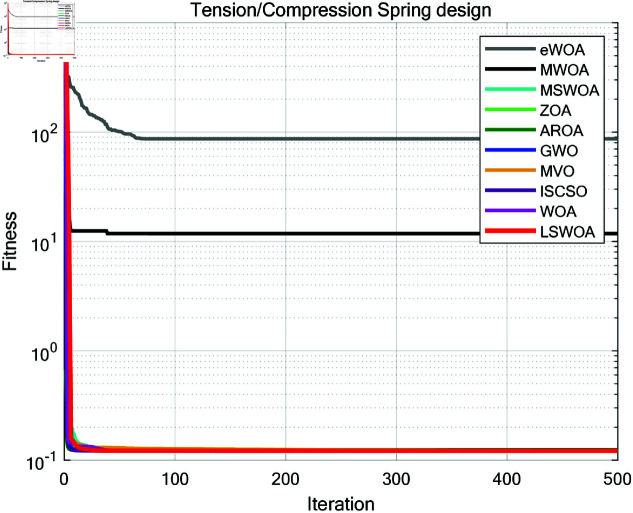
Iteration curves of the algorithms in Tension/Compression Spring design problem.

**Fig 15:**
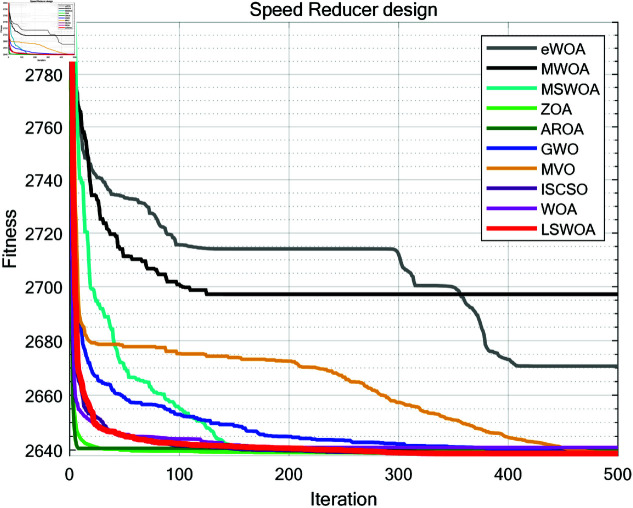
Iteration curves of the algorithms in Speed Reducer design problem.

**Fig 16:**
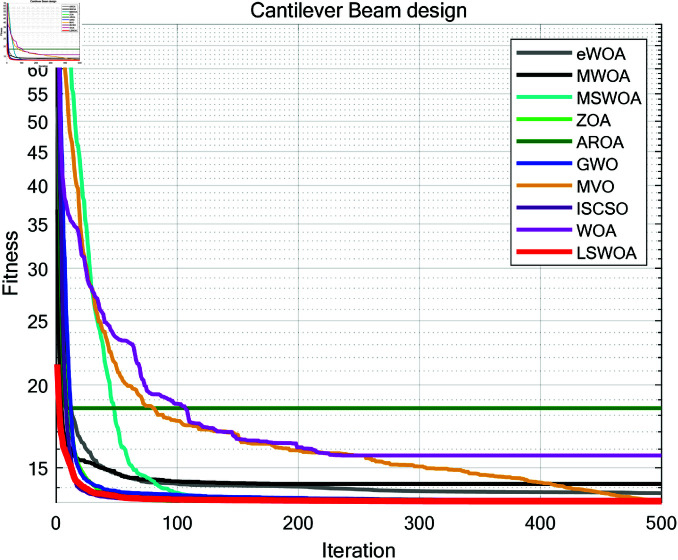
Iteration curves of the algorithms in Cantilever Beam design problem.

**Fig 17:**
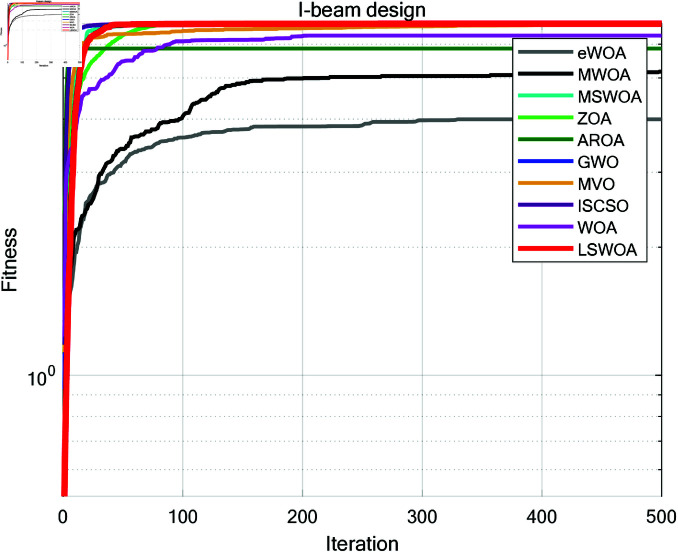
Iteration curves of the algorithms in I-beam design problem.

**Fig 18:**
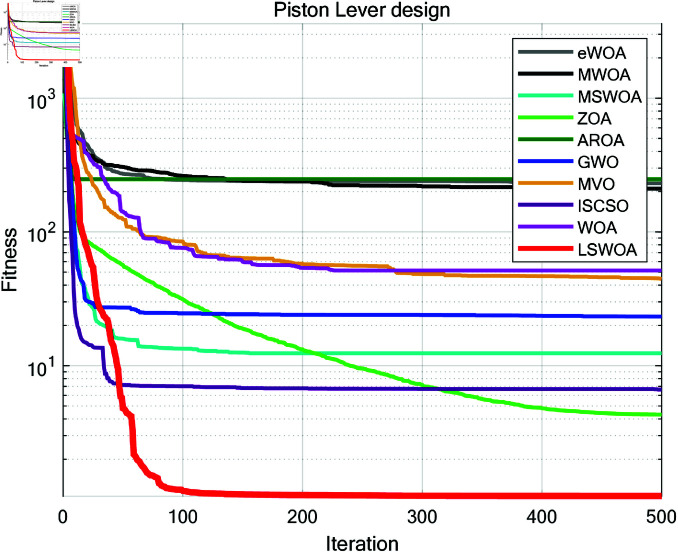
Iteration curves of the algorithms in Piston Lever design problem.

**Fig 19:**
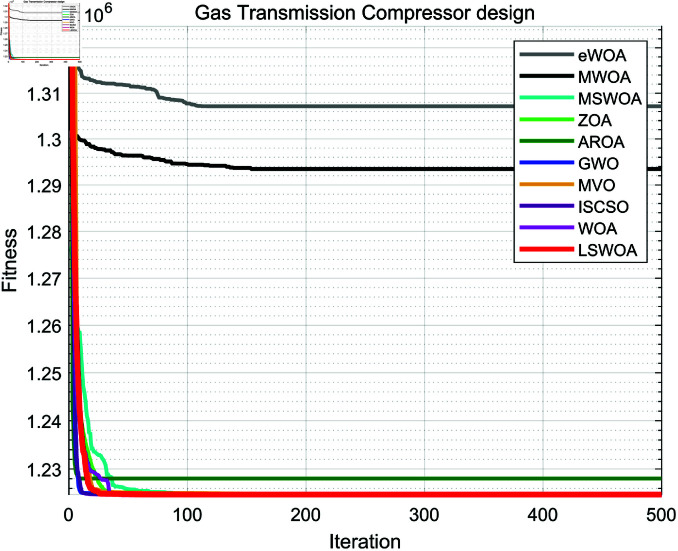
Iteration curves of the algorithms in Gas Transmission System design problem.

A comparative test was conducted between LSWPA and other SOTA algorithms including eWOA [34], MWOA [35], MSWOA [36], Zebra Optimization Algorithm (ZOA) [37], Attraction-Repulsion Optimization Algorithm (AROA) [38], Grey Wolf Optimizer (GWO) [39], Multi-Verse Optimizer (MVO) [40], ISCSO [41] and Whale Optimization Algorithm (WOA) [30]. Parameter settings for each algorithm were shown in Table 3, with the maximum number of iterations *T*=500 and population size *N*=30 uniformly. Each algorithm was run independently 30 times, recording the average fitness value (Ave) and standard deviation (Std) for performance analysis. The experimental results were presented in Figs 13–19 and Table 9.

**Table 9:** Average fitness and standard deviation of each algorithm across the seven engineering design problems. *Ave* indicates average fitness, *Std* indicates standard deviation.

Challenge	Metrics	eWOA	MWOA	MSWOA	ZOA	AROA	GWO	MVO	ISCSO	WOA	LSWOA
Three-bar	Ave	259.807886	260.043760	259.807539	259.805047	259.836984	259.805061	259.805049	259.805056	259.924662	259.805047
Truss	Std	0.003759	0.253007	0.003377	0.000001	0.143568	0.000014	0.000003	0.000015	0.177985	0.000000
Tension/Compression	Ave	86.700869	11.795669	0.121539	0.121523	0.124051	0.121525	0.121530	0.121524	0.121583	0.121522
Spring	Std	264.070383	46.538393	0.000015	0.000001	0.010405	0.000004	0.000008	0.000002	0.000330	0.000000
Speed Reducer	Ave	2670.631503	2697.167015	2639.454262	2638.822181	2640.622447	2638.848863	2638.895325	2638.863072	2640.889342	2638.819849
	Std	60.449630	36.491408	1.141855	0.005039	2.315562	0.034872	0.219400	0.049038	6.314316	0.000070
Cantilever	Ave	13.735503	14.181083	13.416426	13.360305	18.453342	13.360428	13.365048	13.360835	15.656425	13.360259
Beam	Std	0.248505	0.537579	0.041812	0.000052	2.881243	0.000083	0.003419	0.000496	1.644459	0.000000
I-beam	Ave	3.995165	5.167829	6.699097	6.702938	5.859136	6.702773	6.701155	6.702880	6.283696	6.703048
	Std	0.949186	0.995891	0.003601	0.000110	1.093767	0.000277	0.002165	0.000150	0.394099	0.000000
Piston Lever	Ave	231.148191	210.387943	12.387686	4.317819	247.804347	23.276078	44.544733	6.620873	51.509146	1.062983
	Std	111.304972	133.489652	42.967002	7.471606	156.394303	57.611166	99.451828	30.463347	117.091689	0.031553
Gas Transmission	Ave	1307135.966990	1293452.222601	1224747.707182	1224745.937658	1228055.070930	1224745.965924	1224745.992413	1224745.951448	1224745.937231	1224745.937222
System	Std	22396.093418	28729.425559	2.346735	0.000703	11419.629220	0.034177	0.061026	0.013662	0.000021	0.000000

**Table 10:** Standard benchmark functions [42].

Function	Function’s Name	Type	Dimension (Dim)	Best Value
F1	Sphere	Uni-modal, Scalable	30/50/100	0
F2	Schwefel’s Problem 2.22	Uni-modal, Scalable	30/50/100	0
F3	Schwefel’s Problem 1.2	Uni-modal, Scalable	30/50/100	0
F4	Schwefel’s Problem 2.21	Uni-modal, Scalable	30/50/100	0
F5	Generalized Rosenbrock’s Function	Uni-modal, Scalable	30/50/100	0
F6	Step Function	Uni-modal, Scalable	30/50/100	0
F7	Quartic Function	Uni-modal, Scalable	30/50/100	0
F8	Generalized Schwefel’s Function	Multi-modal, Scalable	30/50/100	-418.98·Dim
F9	Generalized Rastrigin’s Function	Multi-modal, Scalable	30/50/100	0
F10	Ackley’s Function	Multi-modal, Scalable	30/50/100	0
F11	Generalized Griewank’s Function	Multi-modal, Scalable	30/50/100	0
F12	Generalized Penalized Function 1	Multi-modal, Scalable	30/50/100	0
F13	Generalized Penalized Function 2	Multi-modal, Scalable	30/50/100	0
F14	Shekel’s Foxholes Function	Multi-modal, Unscalable	2	0.998
F15	Kowalik’s Function	Composition, Unscalable	4	0.0003075
F16	Six-Hump Camel-Back Function	Composition, Unscalable	2	-1.0316
F17	Branin Function	Composition, Unscalable	2	0.398
F18	Goldstein-Price Function	Composition, Unscalable	2	3
F19	Hartman’s Function 1	Composition, Unscalable	3	-3.8628
F20	Hartman’s Function 2	Composition, Unscalable	6	-3.32
F21	Shekel’s Function 1	Composition, Unscalable	4	-10.1532
F22	Shekel’s Function 2	Composition, Unscalable	4	-10.4029
F23	Shekel’s Function 3	Composition, Unscalable	4	-10.5364

### 8.1 Three-bar truss design

**Fig 20:**
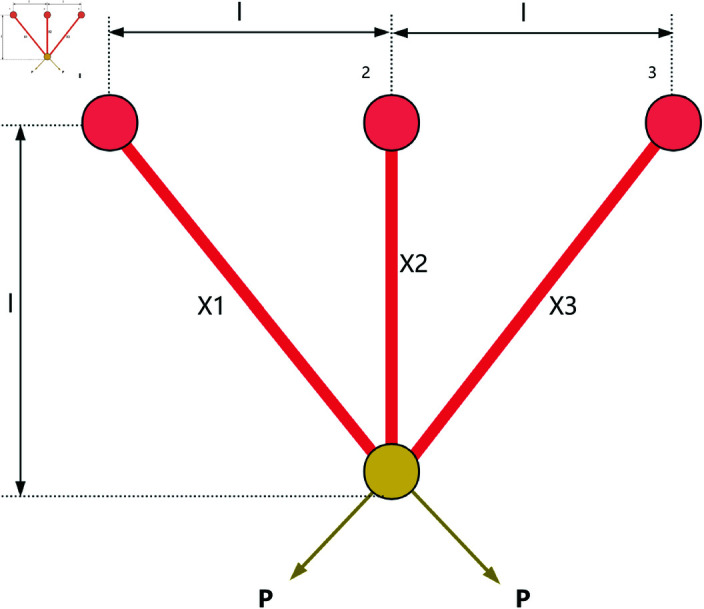
The structure of a three-bar truss.

The three-bar truss is a simple truss structure composed of three bars, as shown in Fig 20. It is commonly used to support concentrated loads and finds wide application in bridge, building, and aerospace engineering. The three-bar truss design problem is a classic structural optimization problem used to study the mechanical behavior of simple structures under external loads. In this problem, the goal is to optimize the cross-sectional area of each bar in the truss to minimize material usage while ensuring the structure meets mechanical performance requirements.

This optimization problem includes a nonlinear objective function, three nonlinear inequality constraints, and two continuous decision variables *x*_1_ and *x*_2_. The objective function for the three-bar truss design problem can be described as,

*Variable:*


$$x=[x_{1},x_{2}]$$

*Minimize:*

$$f(x)=(2\sqrt{2}x_{1}+x_{2})\cdot l$$ (34)

*Subject to:*

$$g_{1}(x)=\frac{\sqrt{2}x_{1}+x_{2}}{\sqrt{2}\cdot x_{1}^{2}+2x_{1}\cdot x_{2}}P-\sigma \leq 0$$ (35)

$$g_{2}(x)=\frac{x_{2}}{\sqrt{2}x_{1}^{2}+2x_{1}\cdot x_{2}}P-\sigma \leq 0$$ (36)

$$g_{3}(x)=\frac{1}{\sqrt{2}x_{2}+x_{1}}P-\sigma \leq 0$$ (37)

*Where:*


$$l=100cm;\;P=2kN/cm^{2};\;\sigma =2kN/cm^{2}$$

*Variable range:*


$$0\leq x_{i}\leq 1,\;i=1,2$$

As shown in Table 9, the stability of LSWOA in the Three-bar Truss design problem significantly surpassed other algorithms, and it achieved the highest optimization accuracy among all algorithms.

### 8.2 Tension/compression spring design

**Fig 21:**
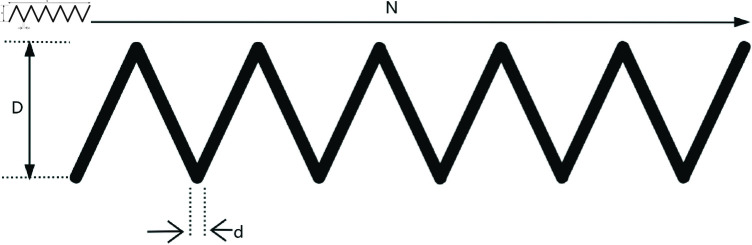
The structure of a tension/compression spring.

The extension/compression spring, as shown in Fig 21, plays a crucial role in modern industry, with widespread applications in fields such as automotive, home appliances, and electronics. Its design optimization not only helps improve product performance and extend service life, but also reduces costs and enhances manufacturing efficiency. Through reasonable design optimization, the spring can achieve optimal performance in dynamic working environments and meet various stringent requirements. The optimization objective of the design problem for the extension/compression spring is the minimization of its mass. The problem needs to be solved under constraints such as shear force, deflection, fluctuation frequency, and outer diameter. There are three design variables in this problem: coil diameter *d*, mean coil diameter *D*, and number of coils *N*. There are also four constraints, *g*_1_ to *g*_4_. The mathematical model of the problem is as follows,

*Variable:*


$$x=[d,D,N]=[x_{1},x_{2},x_{3}]$$

*Minimize:*

$$f(x)=(x_{3}+2)\cdot x_{2}\cdot x_{1}^{2}$$ (38)

*Subject to:*

$$g_{1}(x)=1-\frac{x_{2}^{3}\cdot x_{3}}{71785x_{1}^{4}}\leq 0$$ (39)

$$g_{2}(x)=\frac{4x_{2}^{2}-x_{1}\cdot x_{2}}{12566(x_{2}\cdot x_{1}^{3}-x_{4})}+\frac{1}{5108x_{1}^{2}}-1\leq 0$$ (40)

$$g_{3}(x)=1-\frac{140.45x_{1}}{x_{2}^{2}\cdot x_{3}}\leq 0$$ (41)

$$g_{4}(x)=\frac{x_{1}+x_{2}}{1.5}-1\leq 0$$ (42)

*Variable range:*


$$0.05\leq x_{1}\leq 2,\;0.25\leq x_{2}\leq 1.3,\;2.0\leq x_{3}\leq 15$$

As shown in Table 9, the stability of LSWOA in the Tension/Compression Spring design problem significantly surpassed other algorithms, and it achieved the highest optimization accuracy among all algorithms.

### 8.3 Speed reducer design

**Fig 22:**
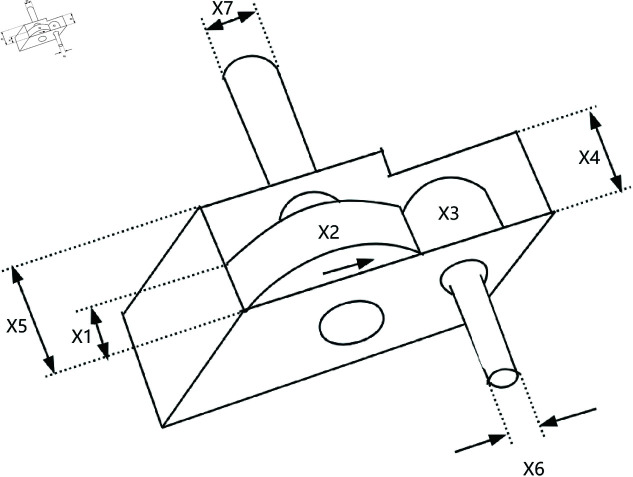
The structure of a speed reducer.

A speed reducer is a mechanical transmission device and one of the key components of a gearbox, shown in Fig 22. It is primarily used to reduce the rotational speed of an electric motor or other power sources while increasing the output torque. The reducer achieves this speed reduction through gears, worm gears, or other transmission mechanisms. It is typically applied in situations where there is a need to decrease the rotational speed, increase torque, or adjust the direction of motion.

In the optimization design of a reducer, the goal is to minimize the weight of the reducer. This problem involves seven variables, which are as follows: the width of the gear teeth *x*_1_, the gear module *x*_2_, the number of teeth on the small gear *x*_3_, the length of the first shaft between the bearings *x*_4_, the length of the second shaft between the bearings *x*_5_, the diameter of the first shaft *x*_6_, and the diameter of the second shaft *x*_7_. Furthermore, this problem also involves eleven constraints, *g*_1_ to *g*_11_. The mathematical formulation of the problem is as follows,

*Variable:*


$$x=[x_{1},x_{2},x_{3},x_{4},x_{5},x_{6},x_{7}]$$

*Minimize:*

y=f(x) (43)

*Subject to:*

$$g_{1}=\frac{27}{x_{1}\cdot x_{2}^{2}\cdot x_{3}}-1\leq 0;$$ (44)

$$g_{2}=\frac{397.5}{x_{1}\cdot x_{2}^{2}\cdot x_{3}^{2}}-1\leq 0;$$ (45)

$$g_{3}=\frac{1.93x_{4}^{3}}{x_{2}\cdot x_{6}^{4}\cdot x_{3}}-1\leq 0;$$ (46)

$$g_{4}=\frac{1.93x_{5}^{3}}{x_{2}\cdot x_{7}^{4}\cdot x_{3}}-1\leq 0;$$ (47)

$$g_{5}=\frac{\sqrt{16.91\cdot 10^{6}+{\left(\frac{745x_{4}}{x_{2}\cdot x_{3}}\right)}^{2}}}{110x_{6}^{3}}-1\leq 0;$$ (48)

$$g_{6}=\frac{\sqrt{157.5\cdot 10^{6}+{\left(\frac{745x_{4}}{x_{2}\cdot x_{3}}\right)}^{2}}}{85x_{7}^{3}}-1\leq 0;$$ (49)

$$g_{7}=\frac{x_{2}\cdot x_{3}}{40}-1\leq 0;$$ (50)

$$g_{8}=\frac{5x_{2}}{x_{1}}-1\leq 0;$$ (51)

$$g_{9}=\frac{x_{1}}{12x_{2}}-1\leq 0;$$ (52)

$$g_{10}=\frac{1.5x_{6}+1.9}{x_{4}}-1\leq 0;$$ (53)

$$g_{11}=\frac{1.1x_{7}+1.9}{x_{5}}-1\leq 0;$$ (54)

*Variable range:*


$$2.6\leq x_{1}\leq 3.6;\;0.7\leq x_{2}\leq 0.8;\;17\leq x_{3}\leq 28;\;7.3\leq x_{4}\leq 8.3;$$

$$7.3\leq x_{5}\leq 8.3;\;2.9\leq x_{6}\leq 3.9;\;5\leq x_{7}\leq 5.5;$$

As shown in Table 9, the stability of LSWOA in the Speed Reducer design problem significantly surpassed other algorithms, and it achieved the highest optimization accuracy among all algorithms.

### 8.4 Cantilever beam design

**Fig 23:**
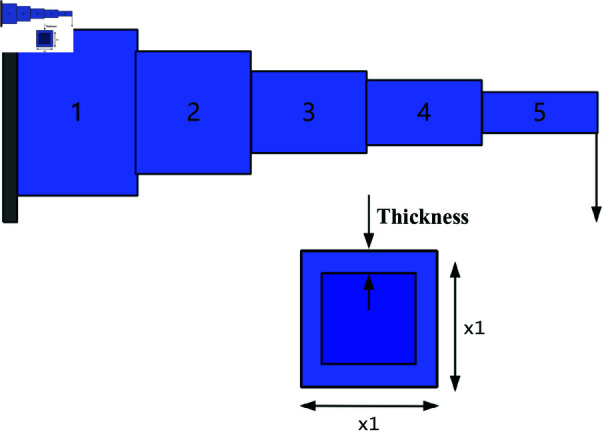
The structure of a cantilever beam.

A cantilever beam is a common structural form, fixed at one end and free at the other, as shown in Fig 23. The cantilever beam design problem is a classic engineering structural optimization problem, with the objective of minimizing material usage or beam weight while satisfying constraints on strength, stability, and other factors. This optimization problem is widely used in civil engineering, mechanical design, and aerospace fields.

The cantilever beam consists of five hollow square cross-section units. As shown in Fig 23, each unit is defined by one variable, and the thickness is constant. Therefore, the design problem includes five structural parameters, which correspond to five decision variables, denoted as *s*_1_, *s*_2_, *s*_3_, *s*_4_, *s*_5_. The objective function for the cantilever beam design problem can be expressed as:

*Variable:*


$$x=[s_{1},s_{2},s_{3},s_{4},s_{5}]=[x_{1},x_{2},x_{3},x_{4},x_{5}]$$

*Minimize:*

$$f(x)=0.0624(x_{1}+x_{2}+x_{3}+x_{4}+x_{5})$$ (55)

*Subject to:*

$$g(x)=\frac{61}{x_{1}^{3}}+\frac{37}{x_{2}^{3}}+\frac{19}{x_{3}^{3}}+\frac{7}{x_{4}^{3}}+\frac{1}{x_{5}^{3}}-1\leq 0$$ (56)

*Variable range:*


$$0.01\leq x_{i}\leq 100,\;i=1,2,3,4,5$$

As shown in Table 9, the stability of LSWOA in the Cantilever Beam design problem significantly surpassed other algorithms, and it achieved the highest optimization accuracy among all algorithms.

### 8.5 I-beam design

**Fig 24:**
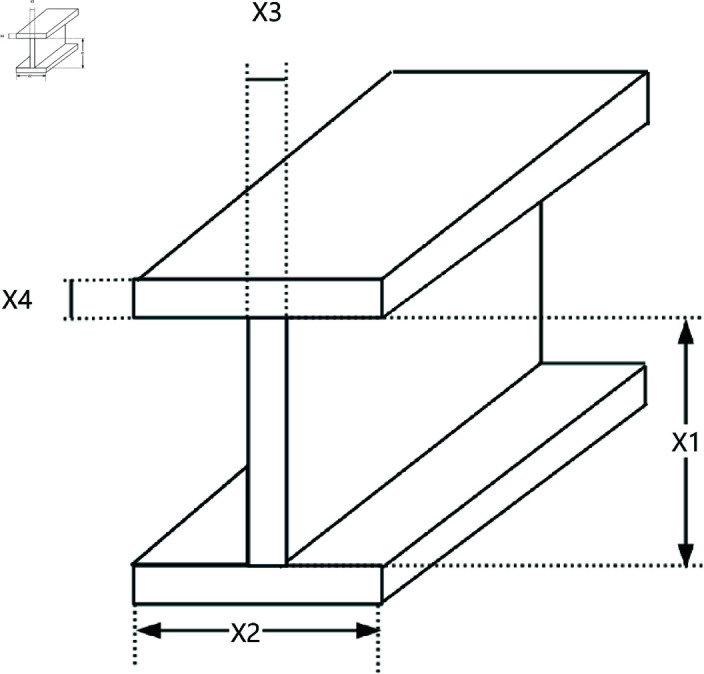
The structure of an I-beam.

An I-beam, named for its cross-sectional shape resembling the letter ’I’, is a type of steel with high strength and low self-weight. It is widely used in various engineering structures. Its superior mechanical properties make it applicable in multiple fields, particularly in structures subjected to bending moments and axial forces. The objective of I-beam design optimization is to select the geometric parameters of the I-beam (such as width, height, thickness, etc.) in a way that maximizes its performance. This typically involves maximizing its load-bearing capacity, minimizing material usage, controlling structural deformations, and reducing costs. Optimizing I-beam design in engineering can enhance the safety, economy, and efficiency of structures. As shown in Fig 24, the I-beam design optimization problem involves four variables (*x*_1_, *x*_2_, *x*_3_ and *x*_4_) and two constraints (*g*_1_ and *g*_2_). *x*_1_, *x*_2_, *x*_3_ and *x*_4_ represent the web height, flange width, web thickness, and flange thickness of the I-beam, respectively. The objective function for the I-beam design problem can be described as.

*Variable:*


$$x=[x_{1},x_{2},x_{3},x_{4}]$$

*Maximize:*

$$f(x)=\frac{5000}{\frac{x_{3}\cdot (x_{1}-2x_{4}{)}^{3}}{12}+\frac{x_{2}\cdot x_{4}^{3}}{6}+2x_{2}\cdot x_{4}{\left(\frac{x_{1}-x_{4}}{2}\right)}^{2}}$$ (57)

*Subject to:*

$$g_{1}(x)=2x_{2}\cdot x_{3}+x_{3}\cdot (x_{1}-2x_{4})-300\leq 0;$$ (58)

$$g_{2}(x)=\frac{18\cdot 10^{4}x_{1}}{x_{3}{\left(x_{1}-2x_{4}\right)}^{3}+2x_{2}\cdot x_{3}\left(4x_{4}^{2}+3x_{1}\cdot (x_{1}-2x_{4})\right)}\leq 0$$ (59)

*Variable range:*


$$10\leq x_{1}\leq 80;\;10\leq x_{2}\leq 50;\;0.9\leq x_{3}\leq 5;\;0.9\leq x_{4}\leq 5;$$

As shown in Table 9, LSWOA significantly outperformed the other algorithms in terms of both optimization accuracy and stability for the I-beam design problem. This demonstrated that LSWOA had superior solving capabilities when handling this type of problem.

### 8.6 Piston lever design

**Fig 25:**
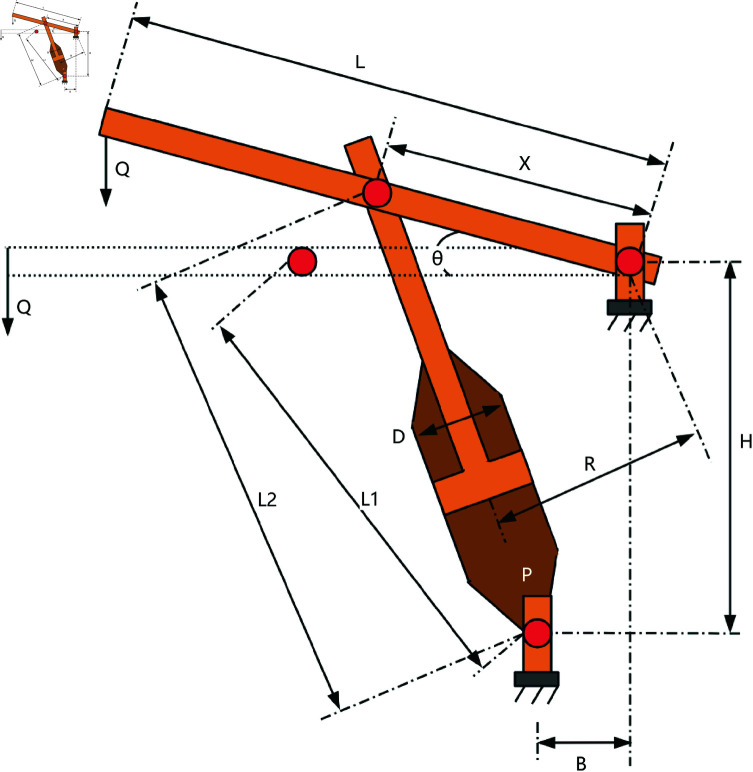
The structure of a piston lever.

A piston lever is a typical mechanical structure, as shown in Fig 25, and its design problem is classified as a classical engineering optimization problem. It involves the adjustment of multiple geometric and mechanical parameters with the aim of minimizing material usage or structural weight while satisfying constraints such as strength and stability. This optimization seeks to achieve a balance between economic efficiency and structural performance and is widely applied in mechanical engineering, vehicle design, and other industrial scenarios, especially in lightweight and efficient design of moving components.

**Fig 26:**
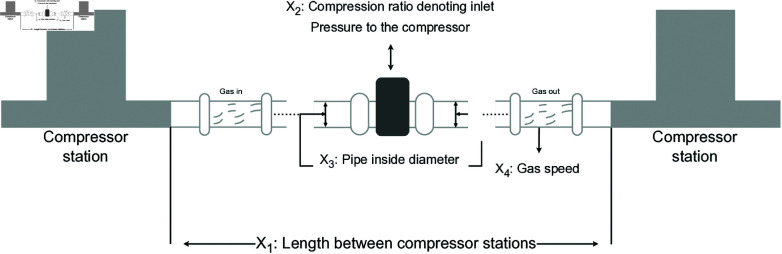
The structure of a gas transmission system.

In the Piston Lever design problem, the objective is to minimize the total material consumption of the Piston Lever while ensuring that the structural strength and performance meet design requirements. The geometric structure of the Piston Lever is defined by multiple design parameters that describe the relationships between its key dimensions. In this problem, the Piston Lever consists of multiple structural parts with the following important characteristics: one end is fixed, while the other end bears an applied force; its mechanical properties are influenced by geometric features such as radius and length; and the design of each geometric part is controlled by decision variables. From the geometric relationships, the meanings of variables *x*_1_ to *x*_4_ are as follows: *x*_1_ and *x*_2_ are Primary length and width parameters of the geometric structure, which govern the overall lever arm. *x*_3_ is cross-sectional radius at the point of force application, affecting force distribution. *x*_4_ is Geometric dimension related to the support point.

The objective function for the Piston Lever design problem can be described as,

*Variable:*


$$x=[x_{1},x_{2},x_{3},x_{4}]$$

*Minimize:*

$$f(x)=0.25\pi x_{3}^{2}(L_{2}-L_{1})$$ (60)

*Subject to:*

$$g_{1}(x)=QL\cos\theta -RF\leq 0;$$ (61)

$$g_{2}(x)=Q(L-x_{4})-M_{\max}\leq 0;$$ (62)

$$g_{3}(x)=1.2(L_{2}-L_{1})-L_{1}\leq 0;$$ (63)

$$g_{4}(x)=\frac{x_{3}}{2}-x_{2}\leq 0;$$ (64)

*Variable range:*


$$0.05\leq x_{1}\leq 500;\;0.05\leq x_{2}\leq 500;\;0.05\leq x_{4}\leq 500;\;0.05\leq x_{3}\leq 120;$$

*Where:*


$$Q=10000;\;P=1500;\;L=240;\;M_{\max}=1.8\times 10^{6};$$

$$L_{1}=\sqrt{(x_{4}-x_{2}{)}^{2}+x_{1}^{2}};\;L_{2}=\sqrt{(x_{4}\sin\theta +x_{1}{)}^{2}+(x_{2}-x_{4}\cos\theta {)}^{2}};$$

$$R=\frac{\left|-x_{4}(x_{4}\sin\theta +x_{1})+x_{1}(x_{2}-x_{4}\cos\theta )\right|}{\sqrt{(x_{4}-x_{2}{)}^{2}+x_{1}^{2}}};$$

$$F=0.25\pi Px_{3}^{2};$$

As shown in Table 9, in the Piston Lever design problem, LSWOA demonstrated significantly superior optimization accuracy and stability compared to other algorithms. This indicated that LSWOA had a substantial advantage in handling such problems.

### 8.7 Gas transmission system design

The gas transmission system is a crucial component of the modern energy supply chain, widely used in various industries, urban natural gas supply, and multinational energy transportation. Since the transportation of natural gas relies on Gas Transmission Compressors and pipeline networks, the design optimization of these devices is essential to ensuring energy transmission efficiency and reducing energy waste. The objective of the Gas Transmission Compressor optimization problem is to design and optimize the parameters of the natural gas transmission compressor, so that the compressor can deliver optimal performance under different working conditions, reduce energy consumption, extend service life, and minimize costs. The Gas Transmission Compressor optimization problem involves four design variables and one constraint. The meanings of the variables *x*_1_ to *x*_4_ are: *x*_1_ indicates the length between compressor stations; *x*_2_ indicates the compression ratio denoting inlet pressure to the compressor; *x*_3_ indicates the pipe inside diameter; *x*_4_ indicates the gas speed on the output side. The mathematical modeling of the Gas Transmission Compressor optimization problem is as follows:

*Variable:*


$$x=[x_{1},x_{2},x_{3},x_{4}]$$

*Minimize:*

$$y=8.61\cdot 10^{5}x_{1}^{\frac{1}{2}}x_{2}x_{3}^{-\frac{2}{3}}x_{4}^{-\frac{1}{2}}+3.69\cdot 10^{4}x_{3}+7.72\cdot 10^{8}x_{1}^{-1}x_{2}^{0.219}-765.43\cdot 10^{6}x_{1}^{-1}$$ (65)

*Subject to:*

$$g=x_{4}x_{2}^{-2}+x_{2}^{-2}-1\leq 0;$$ (66)

*Variable range:*


$$20<x_{1}<50;\;1<x_{2}<10;\;20<x_{3}<45;\;0.1<x_{4}<60$$

As shown in Table 9, LSWOA significantly outperformed the other algorithms in terms of both optimization accuracy and stability for the Gas Transmission System design problem. This demonstrated that LSWOA had superior solving capabilities when handling this type of problem.

In summary, LSWOA demonstrated exceptional solution accuracy and stability in the seven engineering design optimization challenges: Three-bar Truss, Tension/Compression Spring, Speed Reducer, Cantilever Beam, I-beam, Piston Lever, and Gas Transmission System design. LSWOA’s solving capability in engineering design optimization far surpassed that of WOA and numerous SOTA algorithms. This demonstrated the advantages of LSWOA in the field of engineering design optimization, providing a potential application for WOA in this domain.

## 9 Conclusion

LSWOA introduced Good Nodes Set initialization to generate a uniformly distributed population, adopted a newly designed Distance-Guided Prey Searching strategy and a Spiral Encircling Prey strategy, and incorporated Levy flight and inertia weight ω to enhance the Spiral Updating strategy. We also redesigned the update method of convergence factor *a* to better balance global exploration and local exploitation. LSWOA effectively balanced exploration and exploitation during optimization, achieving high convergence accuracy, fast convergence and maintaining population diversity.

Five distinct tests were conducted to verify the effectiveness of the LSWOA improvements, including: (a) a parameter sensitivity analysis experiment for choosing the optimal *k*_1_ and *k*_2_ for the proposed ω and convergence factor *a* respectively, for better balancing the exploration and exploitation; (b) a ualitative analysis experiment was conducted to examine the search behavior of LSWOA across different functions, the ratio of exploitation-exploration, and population diversity; (c) an ablation study of removing different improvement strategies from LSWOA for comparison against the complete LSWOA, ordering to verify the effectiveness of each strategy individually; (d) comparing LSWOA with different SOTA metaheuristic algorithms on classical benchmark functions to demonstrate the effectiveness of LSWOA; (e) comparing LSWOA with different metaheuristic algorithms on classical benchmark functions in higher dimensions of 50 and 100 to demonstrate LSWOA’s effectiveness on solving higher dimensional problems. And finally performing engineering optimization optimization tests on seven engineering design problems to validate LSWOA’s practical applicability. LSWOA offered new insights for the application of WOA in engineering design.

Alghough LSWOA has a fast convergence rate and can effectively balance exploration and exploitation. However, it struggles when dealing with ultra-large-scale mathematical optimization problems. In the future, we will try to enhance LSWOA for ultra-large-scale mathematical optimization problems. Meanwhile, future work will include rigorous testing of manufacturing prototypes, validation in real-world scenarios, and consideration of real-world constraints to enhance the reliability and effectiveness of the optimization process, aiming for more reliable and effective mechanical designs. Finally, LSWOA is recommended as a tool for design, simulation, and manufacturing in line with contemporary industrial demands. We also plan to investigate further applications of LSWOA, including path planning, multi-objective problems, constrained optimization problems, and parameter optimization.

## Appendix A. Standard benchmark functions

To support the experimental study in this paper, we used the Standard Benchmark Functions. The relevant data has been uploaded to Figshare, and the link for the specific modeling of Standard Benchmark Functions (*D*=30) is: https://doi.org/10.6084/m9.figshare.28440863, for reference and further analysis by the readers [42].
